# Effects of Glucose and Fructose on Production Traits, Organ Weights and Metabolomic Indices in Rats on Different Energy and Nutrient Dense Diets

**DOI:** 10.3390/nu17172746

**Published:** 2025-08-25

**Authors:** József Szabó, Gergely Maróti, Norbert Solymosi, Emese Andrásofszky, András Bersényi, Geza Bruckner, István Hullár

**Affiliations:** 1 Department of Animal Nutrition and Clinical Dietetics, Institute for Animal Breeding, Nutrition and Laboratory Animal Science, University of Veterinary Medicine, P.O. Box 2, H-1400 Budapest, Hungary; emese.andrasofszky@univet.hu (E.A.); andras.bersenyi@univet.hu (A.B.); istvan.hullar@univet.hu (I.H.); 2 Institute of Plant Biology, HUN-REN Biological Research Center, H-6726 Szeged, Hungary; marotig@seqomics.hu; 3 Centre for Bioinformatics, University of Veterinary Medicine, P.O. Box 2, H-1400 Budapest, Hungary; solymosi.norbert@gmail.com; 4 Department of Athletic Training and Clinical Nutrition, University of Kentucky, Lexington, KY 40536, USA; gezabruckner0@gmail.com

**Keywords:** body weight, feed conversion, glucose, fructose, lard, liver, cholesterol, triglyceride, insulin, glucagon, leptin

## Abstract

The objectives of this study were to determine the dose effect/s of glucose (G) and fructose (F) at different energy densities (ED) of diets on feed intake, body and organ weights, chemical composition of liver, feed conversion, and metabolomic indices (enzymes and hormones). Methods: Seventy-two 10-week-old male Wistar SPF rats were divided into 9 dietary groups and housed individually in metabolic cages. The control group was on a carbohydrate-free high lard diet (L), and for the other 8 treatment groups, the L content of the control diet was gradually replaced by G or F to decrease the dietary ED, in such a way that the nutrients (protein, minerals and vitamins) to energy ratio of the feeds remained constant. These experimental diets were fed to rats for 28 days. Feed intake and body weight were measured twice weekly. On the 28th day of the experiment, the rats were euthanized, and blood and organ samples were collected for further tests. Results and conclusions: The effects of F and G on twenty-six parameters were measured at different EDs of diets. Significant specific F effects (SFE) over the rats on G diets were found in case of feed intake (statistics with pooled data of feed intake (Fi) showed ~7% more feed intake of F rats: 10.8, 6.4, 9.5 and 2.0% at 5.28, 4.70, 4.23 and 3.85 kcal/g ED, respectively); body weight gain (the relation is polynomial; 8.0, 10.3, 0.1, and −10.2% at 5.28, 4.70, 4.23, and 3.85 kcal/g ED; it related to the weight change of viscera: liver, kidney and RWAT); liver fat (3.98, 21.42, 49.20 and 11.05% at 5.28, 4.70, 4.23, and 3.85 kcal/g ED, respectively); serum triglyceride (the relation is polynomial; 63.2, 88.1, 79.2 and 42.6% at 5.28, 4.73, 4.23, and 3.85 kcal/g ED, respectively); serum glucagon (−1.2, 380.2, 248.3 and 74.7% at 5.28, 4.70, 4.23, and 3.85 kcal/g ED, respectively), and serum leptin (9.59, 30.53, 72.64, and −46.49% at 5.28, 4.70, 4.23, and 3.85 kcal/g ED, respectively). An important conclusion is that in several cases, the effects of F and G were similar in the direction of change, but the magnitude of the effects was different. In case of feed conversion rate, there was no difference between the effect of G and F, however it is important to note that the higher the dietary energy and nutrient density, the better the feed conversion rate (FCR); The potential mechanism(s) of effect for each parameter is discussed and, where appropriate, the clinical relevance of the data compared to the known literature.

## 1. Introduction

It is generally accepted that excessive consumption of energy, i.e., fat [1] and or carbohydrates [2], is an important factor in the pathology of obesity and metabolic syndrome [3]. In studies about the pathology of obesity, questions have been raised about the significance of dietary energy density [4], the satiating power of the foods [5], and the specific effects of the monosaccharides, i.e., glucose (G) and fructose (F). Some researchers suggest that humans have a weak innate ability to recognize energy-dense foods and fail to compensate appropriately to maintain energy balance [6].

The energy density (ED) and nutrient density of food are two different concepts. ED refers to the number of calories per gram of diet. The consumption of high-ED foods results in bodyweight gain and plays a significant role in the development of obesity.

Contrary to this, nutrient density refers to the amount of nutrients relative to the calories in food. High-nutrient-dense foods contain more nutrients relative to the calories and can be used in weight management.

Carbohydrates and fats are energy-rich nutrients. G and F are simple sugars and may have different effects on the metabolism that can contribute to obesity.

Glucose is absorbed from the small intestine and mainly escapes first-pass removal by the liver [7] and can be metabolized by almost all cells in the body. Its primary metabolic fates are the immediate energy production (ATP) through glycolysis and the citric acid cycle, storage as glycogen, and conversion to other molecules like fatty acids and amino acids. Glucose is also used in the pentose phosphate pathway and other minor pathways.

Fructose is absorbed from the small intestine. The bulk of absorbed F is metabolized almost entirely by the liver enzyme fructokinase. It phosphorylates fructose in the hepatocytes to fructose-1-phosphate, which can bypass a key regulatory step in glycolysis and is then converted into triose-phosphates. It is subsequently oxidized into G and lactate [8]. A smaller quantity of triose-phosphate is converted to triacylglycerol by de novo lipogenesis [9], which is associated with the adverse metabolic effect of F, e.g., fat accumulation in the liver and obesity.

High fat intake is another strong contributor to the epidemic of metabolic syndrome-related diseases [10], such as obesity, diabetes, heart failure, and high blood pressure. The optimal carbohydrate–fat ratio is not clear.

In our previous paper [11], we reported the specific effects of well-balanced diets containing different energy sources such as starch (St), G, F, or lard (L) on different nutritional, metabolomic indices, and microbiome parameters of rats. We found the following results: Rats on the L diet consumed less feed, more energy, and gained more than the animals on the St diet, indicating that, in addition to higher energy intake, better feed utilization is a key factor in the obesogenic effect of diets of high nutrient and energy density.

The G, F, and L diets significantly increased the lipid content of the liver, suggesting that lipid accumulation in the liver is not an F-specific process. Relative to the St control, specific G effects were noted as significantly decreasing serum glucagon concentration and glucagon/leptin ratio with increasing serum leptin concentration; specific F effects were noted as increased weights of the kidney, spleen, epididymal fat, and the decreased weight of retroperitoneal fat while, the weight of the eviscerated body did not show any significant different from the St control. Significant F effects were also noted as lower immune response, as well as the increased insulin, glucagon, and decreased leptin levels. We suggested a mild insulin resistance and catabolic metabolism in F rats. Specific L effects were noted by the decreased insulin and increased glucagon and leptin levels.

Therefore, the aim of the current study was to determine the metabolic effects of F relative to the isocaloric G-containing diets at different dietary energy levels with a constant nutrient (protein, minerals, and vitamins) to energy ratio.

## 2. Materials and Methods

The experimental protocol was approved by the Scientific Ethics Council for Animal Experiments established by the Hungarian Government (No. 465/2013/VII. 24.) and the Animal Protection and Welfare Committee of the University of Veterinary Medicine, Budapest, Hungary (Project number: 22.1/5003/2010; 20/2015).

### 2.1. Animals

Seventy-two, 10-week-old male, Wistar, SPF rats, weighing 200–275 g, were divided into 9 groups (*n* = 8/group) with similar average starting body weights (avg. 241.71 ± 17.66 g). Animals were housed individually in wire-bottom cages [light cycle 12 h on (7 a.m.–7 p.m.) 12 h off (7 p.m.–7 a.m.)]. The rats received the experimental diets for 28 days. Water and feed were provided ad libitum during the experiment. Treatment groups and the composition of diets are indicated in Table 1.

### 2.2. Design of Diets

A carbohydrate-free diet was prepared by substituting L calories for carbohydrates in the AIN-93G formula for growing rats (carbohydrate-free control); therefore, the energy added with L was equal to the energy removed by carbohydrates.

In the other treatment groups, the L contents of this modified diet were gradually replaced by G or F to balance the L energy removed. The individual components of the created formulas were expressed as a percentage of the total weights of the mixture (Table 1).

We have one high ED carbohydrate free (L_6.03_ diet; the subscription indicates the energy density of the diet) and four gradually decreasing ED diets, in which either G or F (GL_5.28_, GL_4.70_, GL_4.23_, G_3.85_ or FL_5.28_, FL_4.70_, FL_4.23_, F_3.85_) were the carbohydrate, with constant nutrients (e.g., protein, minerals, vitamins) to energy ratio (Table 1). The composition of the lowest ED G_3.85_ diet represents the recommended energy and nutrient requirement levels of the growing rats.

### 2.3. Blood, Plasma, and Serum Samples

Blood, plasma, or serum samples were collected in glass tubes (heparinized for plasma), kept on ice, and then centrifuged (4–8 °C, 10 min, 492 RCF) to obtain the plasma or serum fraction.

### 2.4. Tested Parameters

The following parameters were investigated: feed intake (Fi), body weight (BW) and body weight gain (BWG), eviscerated body weight (EVBW), feed conversion rate (FCR; Feed/Gain), organ weights (liver, kidney, spleen), epididymal white adipose tissue (EWAT), retroperitoneal white adipose tissue (RWAT), serum glucose, fructosamine, thotal cholesterol (STCh), triglyceride (STG), lactate dehydrogenase (LDH), insulin, glucagon, and leptin.

### 2.5. Body Weight (BW), Feed Intake (Fi)

BW and Fi intake were measured three times a week, between 8:00 and 10:00 in the morning.

### 2.6. Organ Weights, Eviscerated Body Weight (EVBW)

On the 28th day of the trial, the rats were anaesthetized with sodium pentobarbital (35 mg/kg BW) and exsanguinated via the caudal vena cava between 8 and 11 a.m. following a sequence that randomized the treatment groups. After the collection of blood samples, the weights of viscera, the EVBW, and the weights of the liver, kidneys, epididymal, and retroperitoneal fat pads were measured.

### 2.7. Biochemical Parameters

The G, STCh, STG, fructosamine concentrations, and LDH activity of the serum samples were determined by an automated fluid chemical analyzer (Olympus 400, Olympus, Hamburg, Germany) in the Diagnostic Laboratory of the University of Veterinary Medicine, Budapest, Hungary.

### 2.8. Hormones

Insulin, glucagon and leptin concentrations of the blood serum were analyzed by a ROCHE Cobas e 411 fully automated immunoassay analyzer, using Rat Leptin ELISA Kit (Sigma, St. Louis, MO, USA, RAB0335), Rat Ins1/Insulin ELISA Kit (Sigma, RAB0904) and Rat Glucagon ELISA Kit (Ray Biotech, 3607 Parkway Ln STE 200, Peachtree Corners, GA, USA, P06883) for serum, plasma and cell culture supernatant, in the Biochemical Laboratory of DRC Ltd. (Drug Research Centre, Balatonfüred, Hungary).

### 2.9. Statistical Analysis

SPSS version 18.0 software (IBM SPSS Statistics, IBM Corporation, Armonk, NY, USA) [12] was used for the statistical analysis, which included ANOVA followed by post hoc LSD and polynomial regression analysis. The yEd graph editor software (version 3.25.1) [13] was used to create networks and for the analysis of correlations and centrality (Weight of Connected Edges).

All parameter pairs were correlated, and the statistic was tested [14]. Below the *p* < 0.05 threshold, we handled the variables as connected independently by the correlation direction. These connections are represented by edges in the graphs, and the nodes are the variables.

Weight centrality measures the weight associated with incoming, outgoing, or all edges of a node.

In our network, those measured parameters with higher centrality scores are more interconnected with the larger network of metabolic compounds that affect x.

In network science, centrality is a measure of the importance of the nodes composing the network. In weighted networks, the degree centrality is calculated as the sum of weights assigned to the node’s direct connections and represents the node strength. It is then based on tie weights and not on the number of ties.

The specific fructose effect (SFE) is determined as the relative difference between the isocaloric FL, F, and GL, G groups. It is calculated by the following formula:

(the mean of FL or F group/the mean of GL or G group × 100) − 100 = % difference.

The experimental protocol met the standard criteria of the Scientific Ethics Committee of Animal Experiments of the University of Veterinary Medicine, Budapest, Hungary.

## 3. Results and Discussion

### 3.1. Diet Formulation

In deciding how to study the dose-dependent effects of glucose (G) or fructose (F) versus of a carbohydrate-free diet, in which lard (L) is the energy source, the problem faces is that replacing L with G or F, changes the energy content of the diets and also the nutrients (e.g., protein, minerals, vitamins) to energy ratio. If we want to maintain the nutrients-to-energy ratio in the feed, then the energy and nutrient densities of the diets will change. Consequently, in this experimental model, the one-variable comparison of the effect of G and F is possible only at the different isocaloric energy levels.

### 3.2. Limitations and Future Perspectives

The limitation of this study is that the used experimental model does not allow for the determination of the specific effect of the changing ED, or for the effect of the different types of monosaccharides as related to the proportion change of G or F to L in diets. However, in those cases where the effect of diets containing G or F differs significantly at one or more ED levels, a so-called specific fructose effect (SFE) can be noted. The SFE is determined as the relative difference between the isocaloric FL, F and GL, G groups. In fact, it can be named as a specific monosaccharide effect too, as we only assume that fructose is responsible for the difference between the two isocaloric monosaccharides. In reality, for this difference, both carbohydrates can be responsible as well as any one of them.

The future perspectives of our present study can be the investigation of the possible interactions among the dietary carbohydrates, gut microbiota, and the metabolomic indices.

### 3.3. Production Measurements

#### 3.3.1. Feed Intake (Fi)

It is generally accepted that the daily Fi is primarily controlled by the energy requirements [15] and hormonal effects [16] of rats. Other factors also play a role, such as the hypothalamic centers, sensory input, visceral sensing, thermogenesis, anorexic peptides, glucose metabolism, and lipid and amino acid metabolic effects.

The effects of energy-dense nutrients, i.e., L, G, or F, on feed and energy intake are controversial.

The high ED high-fat diets increased weight gain, body fat mass, mesenteric adipocytes’ size, adiponectin and leptin plasma levels, and decreased oral G tolerance in rats [17].

Systemic (intra-mesenteric or intrajugular) injection of G did not reduce food intake, but intragastric or intraduodenal infusion of G reduced it [18]. G is known as an indicator of energy status and signals higher brain centers to adjust feeding behavior and energy expenditure. As the G level in the brain rises, the feed intake is suppressed [19].

Contrary to G, F bypasses the rate-limiting step of glycolysis and, by a rapid ATP-requiring reaction, abruptly depletes ATP and leads to a compensatory rise in AMP, and by the AMPK/malonyl-CoA signaling system, it influences the feeding behavior and paradoxically increases Fi [20].

In our previous paper [11], we also proposed a F paradox hypothesis; since excess F decreases the activities of such enzymes as aconitase and GOT in the TCA cycle [21], despite the high cytosolic triose concentration in the hepatocytes, the resynthesis of ADP to ATP is slower or inhibited.

Therefore, energy-depleted hepatocytes act like starving cells, sending signals (uric acid and P_i_ release) to the pancreatic α- and β-cells and to the hypothalamus–pituitary axis, resulting in increased catabolic hormone secretion (glucagon and corticosterone) [22]. It is well documented that appropriate concentrations of naturally secreted corticosteroids have major stimulatory effects on caloric intake [23].

**Figure 1:**
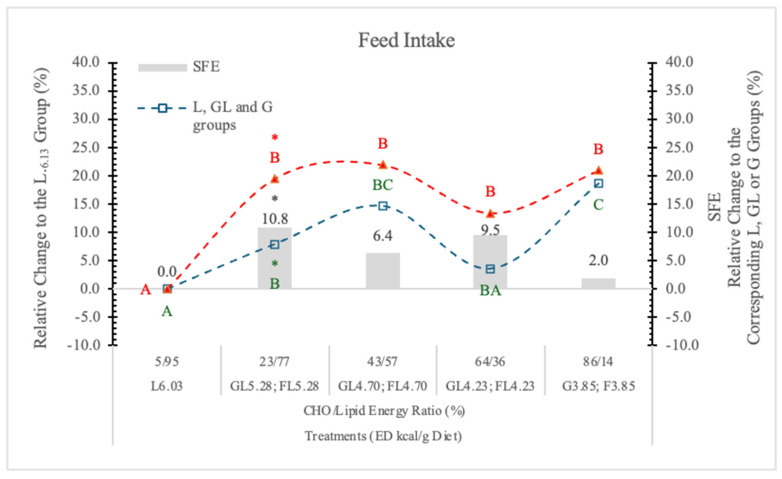
Effect of G and F on the feed intake of rats on different ED diets. L: lard, G: glucose, F: fructose. Different capital letters show a significant difference among the groups consuming diets with the same carbohydrate. The star (*) shows a significant difference between the effect of GL, G or FL, F at the same energy density (ED) level. SFE: specific fructose effect (relative difference between the isocaloric FL, F and GL, G groups).

In the present experiment, the rats on the highest ED, no-carb L_6.03_ diet, ate significantly less (−15.7%) than the animals on lower ED carbohydrate diets (Table 2 and Figure 1). This can be the result of the better taste of the carbohydrate diets compared to the high-fat carb-free feed. Others [24] reported that in rats fed high-energy diets, taste, rather than fat content, is the key factor increasing food intake. The rats on the FL or F diets ate more than their counterparts on isocaloric GL or G formulas. The difference between the effects of the two monosaccharides (G and F) on the appetite was significant only at 5.38 kcal/g dietary ED (Table 2 and Figure 1).

In statistical analysis with pooled data of GL, G, or FL, F groups showed that the difference between two monosaccharides (G and F) on feed intake proves to be significant (*p* < 0.05). This difference can be attributed to the appetite-inhibiting effect of G [19].

Summarizing the data, the most important factor influencing the feed intake is the consumed quantity of the diet, the food content (fullness) of the stomach. A significant appetite-depressing effect can be seen in the case of the high ED carbohydrate-free L_6.03_ diet.

The appetite-suppressing effect of GL and G, relative to the isocaloric FL and F diets, is apparent. Figure 1 shows that on the lowest ED diet (3.85 kcal/g), the difference between G and F in the feed intake of rats is minimal.

#### 3.3.2. Glucose (G) and Fructose (F) Intake

When the data are pooled for the two monosaccharide groups, the significant difference (*p* < 0.05) between them is evident (Table 2).

**Figure 2:**
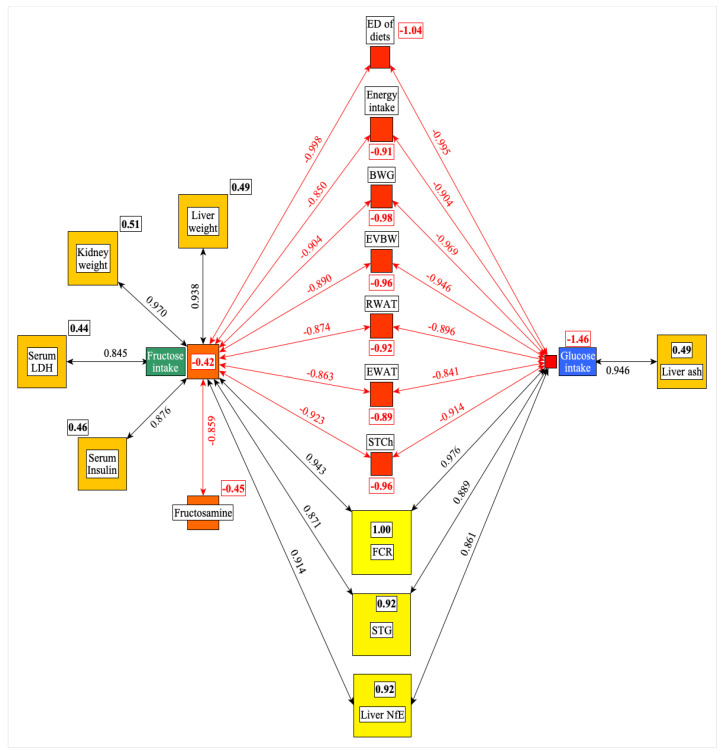
Significant correlations were found among the G or F intake and the other parameters studied. The framed numbers and the colors (yellow: the most central; red: the least central) show the positive or negative weight of a given parameter in the network. ED: energy density; BWG: bodyweight gain; EVBW: eviscerated bodyweight; RWAT: retroperitoneal white adipose tissue; EWAT: epididymal white adipose tissue; STCh: serum total cholesterol; FCR: feed conversion rate; STG: serum triglyceride; NfE: N-free extract; LDH: lactate dehydrogenase.

Figure 2 shows significant correlations among the G or F intake and the other studied parameters. Based on these data, there are more similarities than differences in the effects of dietary G and F on the studied parameters. Both the G and F intakes correlated positively with FCR, STG, liver NFE, and negatively with STCh, EWAT and RWAT, EVBW, BWG, energy intake, and ED of diets. The data also show quantitative differences between the G or F effects, but the direction of their effects is similar.

F intake correlated positively with liver and kidney weight, serum LDH and insulin levels, but negatively with fructosamine, suggesting that the changes of these parameters are the specific result of F intake.

The liver ash content shows a positive correlation with the G intake of rats. The centrality analysis (numbers in small rectangles) indicates that the FCR (feed/gain) and the ED of diets are the most central (positive or negative effect) parameters in this network (Figure 2).

#### 3.3.3. Energy Intake

It has been reported that the effect of dietary energy intake is controversial. In short-term studies, higher ED increased the energy intake more than it decreased the Fi, and in longer-term studies, high ED was more effective at decreasing Fi than at elevating energy intake [25].

**Figure 3:**
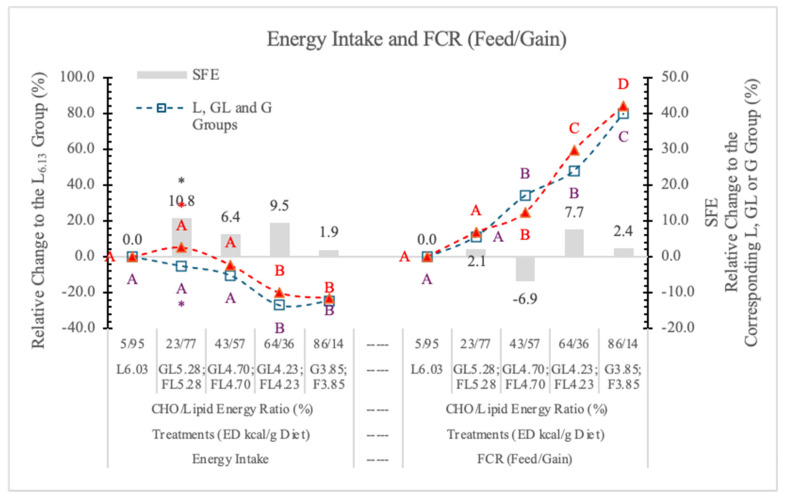
Effect of G or F on the energy intake and feed conversion rate of rats fed different ED diets. L: lard, G: glucose, F: fructose. Different capital letters show a significant difference among the groups consuming diets with the same carbohydrate. The star (*) shows a significant difference between the effect of GL, G or FL, F at the same ED level. SFE: specific fructose effect (relative difference between the isocaloric FL, F and GL, G groups).

In this experiment, the daily energy intake of rats changed according to the Fi and ED of the diets. Compared to the L_6.03_ group, the difference was significant (*p* < 0.05) at 4.23 and 3.85 kcal/g ED in both the G and F groups (Table 2 and Figure 3). The difference between the two monosaccharide (GL and FL) groups was significant (*p* < 0.05) only at 5.28 kcal/g energy density.

The energy intake was affected to a lesser extent by the amount of feed consumed than the ED of the diets; basically, the dietary ED was the determining factor (Figure 3). Statistical analysis with pooled data of GL, G, and FL, F groups showed differences between Gl, G and FL, F groups to be significant at *p* = 0.021. The SFE on the energy intake is small and only significant at 5.28 kcal/g ED (Figure 3).

#### 3.3.4. Bodyweight (BW) and Bodyweight Gain (BWG)

**Figure 4:**
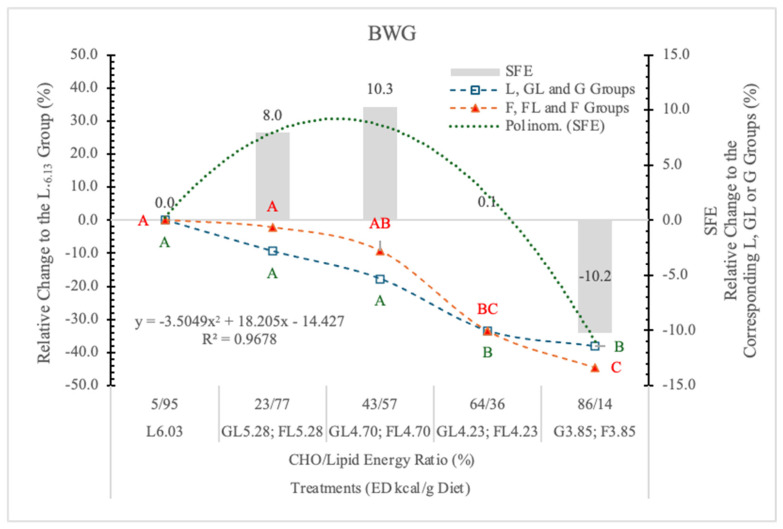
Effect of G or F on the BWG of rats fed different ED diets. Different capital letters show a significant difference among the groups consuming diets with the same carbohydrate. SFE: Specific fructose effect (relative difference between the isocaloric FL, F and GL, G groups).

Relating to the L_6.03_ group, the BW and the BWG of rats on GL, G or FL, F diets decreased in parallel with the change of the energy and nutrient densities of the diets (Table 2 and Figure 4).

The BWG was significantly influenced by the ED of diets, although there was no statistically significant difference between the effect of the two monosaccharides; however, an interesting tendency of specific fructose effect (SFE) can be seen (Figure 4).

The SFE was 10.22% lower at 3.85 kcal/g ED, identical at 4.23 kcal/g ED, and when the energy and nutrient intake was much higher than the maintenance requirements of rats (at 4.70 and 5.28 kcal/g ED), it was 10.3% and 8% more than in the corresponding GL groups. A significant polynomial trend line can be fitted to these data (Figure 4).

Based on the significant difference between the pooled data of the two monosaccharide groups, and the polynomial trendline that can be fitted to the SFE, it appears that the F content and the ED levels of the diets determined the magnitude and direction of F on the BWG.

The negative tendency of SFE at low ED (3.85 kcal/g) is in accordance with the result of Mahmood et al. [26], i.e., that fructose administered in the drinking water decreased the body weight gain of rats compared to water-treated control animals.

No fructose effect on BWG was found when it was substituted for other carbohydrates in diets providing similar calories, and free F at high doses that provided excess calories modestly increased BW, an effect that may be due to the extra calories rather than the fructose [27].

These controversial effects of F on the BW gain can probably be explained by how F is metabolized. At high concentration and low dietary ED, F is rapidly absorbed and phosphorylated mainly in the liver, resulting in energy loss, uric acid formation, and insulin resistance, leading to a number of deleterious effects in the body, especially in the liver.

At a low F concentration, most of the consumed F may be transformed to glucose in the enterocytes [28]. A smaller quantity of dietary F entering the liver may stimulate adipogenesis, glycogen synthesis, and storage. The dietary ED effect of F on BW gain (Figure 4) presents a good explanation for the controversial data in the literature.

#### 3.3.5. Feed Conversion Ratio (FCR; Feed/Gain)

The FCR showed a strong linear negative correlation with the increase of ED of diets, indicating that the higher the ED, the better the feed conversion, and there was no significant difference between the effects of G- or F-containing diets (Figure 3).

This improvement of FCR was parallel with the change of energy and nutrient density of diets, and was not carbohydrate-specific. This phenomenon is important in terms of explaining the obesogenic effect of food.

#### 3.3.6. Eviscerated Body Weight (EVBW)

Relating to the L_6.03_ group, the EVBW of rats on GL, G or FL, F diets decreased in parallel with the energy and nutrient densities of the diets; however, there was no significant difference between the corresponding isocaloric GL, G and FL, F groups (Table 3).

In our earlier four-week-long feeding experiment [11], we found that in isocaloric conditions, when the 65% of starch content of the control diet was replaced with G or F, the EVBW of rats was significantly less (−4.9 and −6.6%, respectively) than in the control animals. In the present situation, when G was the single carbohydrate in the control diets, replacing it with F did not induce a significant change in the EVBW of rats, and there was no significant SFE.

### 3.4. Viscera and Organ Weights

When designing the experiment, our working hypothesis was that F would affect organs that have the GLUT-5 F transporter and KHK-C enzyme; these organs are the small intestine, liver, kidney, and pancreas [29]. The other organs and muscles have KHK-A enzyme, which has a much higher Km than KHK-C; the cells of these organs can metabolize F when its plasma concentration is high, consequently, these organs are less sensitive to the negative effects of F.

#### 3.4.1. Effect of Glucose or Fructose on Visceral Organ Weights

Visceral organs are the soft internal organs of the body, specifically those within the chest (as the heart or lungs) or abdomen (as the liver, pancreas, kidneys, and intestines).

**Figure 5:**
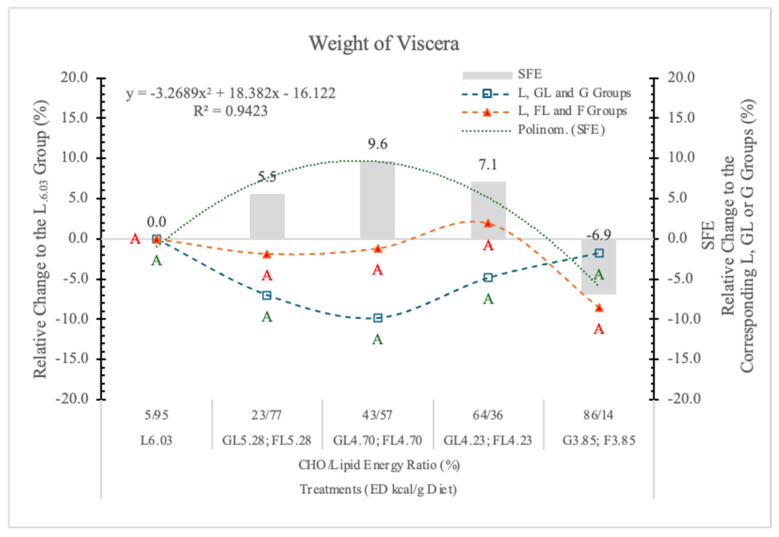
Effect of glucose or fructose on the weight of viscera of rats fed different ED diets. Different capital letters show a significant difference among the groups consuming diets with the same carbohydrate. SFE: specific fructose effect (relative difference between the isocaloric FL, F and GL, G groups).

In this experiment, the SFE was the strongest (9.63%) when the carbohydrate to lipid energy ratio was close to each other (43% to 57%) at 4.73 kcal/g dietary ED level (Table 3 and Figure 5). Under or above this carbohydrate/lipid energy ratio, the SFE was smaller or even negative at the highest dietary F and the lowest dietary ED. A significant polynomial trendline can be fitted to the data of SFE (Table 3 and Figure 5).

We conclude that the SFE on BW gain is mainly due to the weight gain of visceral organs. It is negative at high F content with diets low in ED, or positive when the energy level of diets is higher than the energy requirement of rats. The direction of the effect depends on both the F content and the ED of the diets (Figure 5). The correlation between the BW gain and the weight of visceral organs is significant (r = 0.943; *p* < 0.05).

These results area in agreement with the data of Stanhope et al. [30]; the consumption of F-sweetened beverages increases visceral adiposity and decreases insulin sensitivity in overweight/obese humans.

#### 3.4.2. Epididymal and Retroperitoneal White Adipose Tissues (EWAT and RWAT)

**Figure 6:**
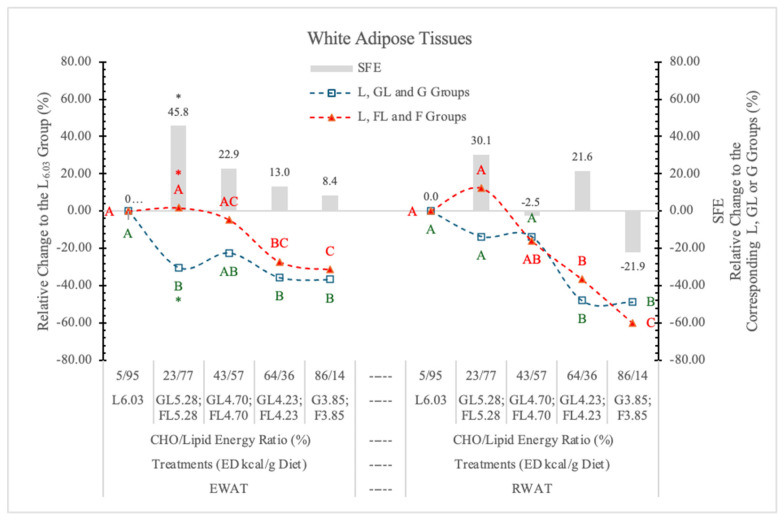
Effect of G or F on the weight of EWAT and RWAT of rats fed different ED diets. Different capital letters show a significant difference among the groups consuming diets with the same carbohydrate. The star (*) shows a significant difference between the effect of GL, G and FL, F at the same ED level. SFE: specific fructose effect (relative difference between the isocaloric FL, F and GL, G groups).

Relative to the highest ED carbohydrate-free L_6.03_ diet, G prevented the higher energy intake-induced weight gain of EWAT, even at low dietary concentration. The same levels of F increased the weight of EWAT parallel with the change of the dietary ED, and at 5.38 kcal/g energy density, the difference between GL and FL groups was statistically significant (*p* < 0.05) (Table 3 and Figure 6). According to the statistical analysis with pooled data of rats on GL, G or FL, F diets, the difference between the two monosaccharide groups was highly significant (*p* = 0.017).

It may be concluded that G, but not F, prevents the effect of the increasing dietary ED on the weight of EWAT.

Contrary to this, the weight of RWAT changed parallel to the dietary ED, independent of the type of monosaccharides, and there were no significant differences between the two monosaccharide groups, neither at the individual ED levels nor for pooled data (Figure 6).

Crescenso et al. [31], in an eight-week-long experiment with rats on an isocaloric high F or control diet, reported significantly increased weight of EWAT and mesenteric white adipose tissues in the high-F group.

Our experimental data show that the high dietary F concentration with low ED (G_3.85_ and F_3.85_ groups) oppositely influence the SFE on EWAT (+8.4%) and RWAT (−21.9%) (Figure 6). The relative difference between the SFE on the two types of adipose tissues was 30.3% (*p* < 0.027). Above this ED level, F increased the weight of both EWAT and RWAT.

In our earlier experiment [11], replacing the starch content of a 3.85 kcal/g ED diet with either G or F, both monosaccharides significantly decreased RWAT, but F was more effective (−27.3%) than G (−6.9%). Others [32] reported that F significantly increased the weight of RWAT over the control values.

The reason for this discrepancy can probably be explained by the difference in the dietary ED levels.

Bjørndal et al. [33] suggest that “adipose tissue metabolism is closely linked to insulin resistance, and differential fat distributions are associated with disorders like hypertension, diabetes, and cardiovascular disease”.

#### 3.4.3. Weight of Liver

Different sugars can have different effects on the weight of the liver, regardless of whether the sugars are consumed in calorically equal amounts. For example, we reported [11] that the replacement of starch with G or F in a rat diet resulted in significantly higher liver mass in both cases (13.9 and 23.9%, respectively) than it was in rats on the starch-containing control diet.

In the present experiment, the effect of F on isocaloric G diets at different ED levels was compared.

**Figure 7:**
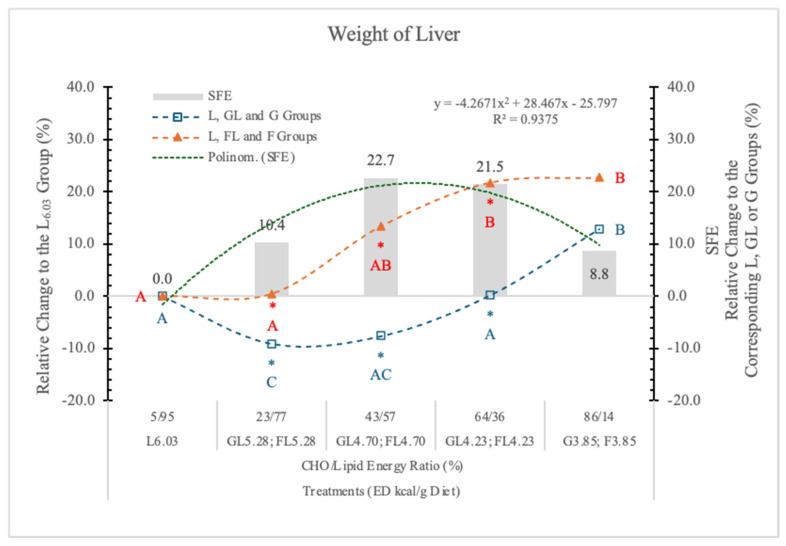
Effect of G or F on the weight of the liver of rats fed different ED diets. Different capital letters show a significant difference among the groups consuming diets with the same carbohydrate. The star (*) shows a significant difference between the effect of GL, G and FL, F at the same ED level. SFE: specific fructose effect (relative difference between the isocaloric FL, F and GL, G groups).

In animals on the G_3.85_ and F_3.85_ diets, the weight of the liver was similar (Table 4, Figure 7). In both the FL and GL dietary groups, the weight of liver followed the decreasing dietary carbohydrate levels, but in the FL groups, it was always significantly higher than in the corresponding GL animals (Table 4 and Figure 7).

These data suggest that the effect of F and G on the weight of the liver depends on the dietary ED and also on the type and quantity of carbohydrate in the diets.

It has been reported [34] that in the pathology of non-alcoholic fatty liver disease (NAFLD), the F consumption plays a significant role in liver pathogenesis. Our experimental data show that the liver of rats on the F-containing diets is significantly larger than that of animals on an isocaloric G-containing diet. The magnitude of this SFE depended on both the dose of F and the energy densities of the diets; a polynomial trendline can be fitted to the data (Figure 7).

F and G usually appear together in the same proportion in our food (e.g., sugar; high-F corn syrup) and in high dietary concentration, both may increase the lipid content of the liver [11], and both can be important factors in the pathophysiology of NAFLD. It was reported that there are significant synergistic effects of F and G on lipoprotein risk factors [35] and net hepatic glycogen synthesis [36].

#### 3.4.4. Weight of Kidney

**Figure 8:**
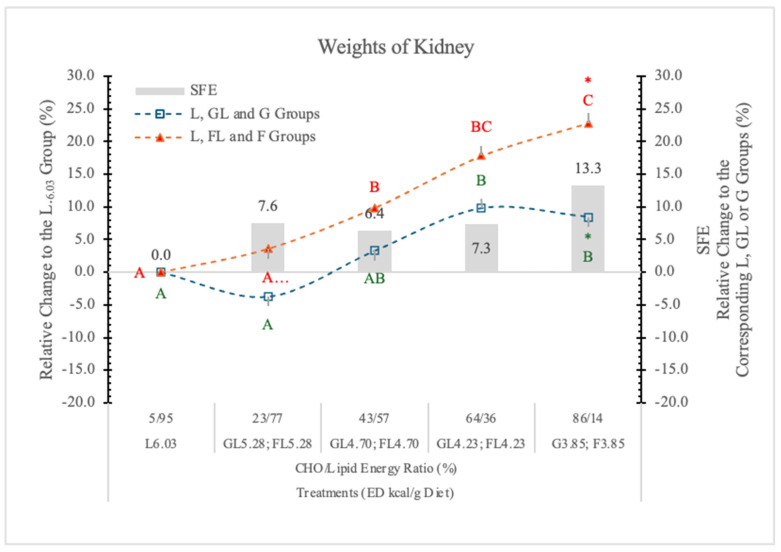
Effect of G or F on the weights of the kidneys of rats fed different ED diets. Different capital letters show a significant difference among the groups consuming diets with the same carbohydrate. The star (*) shows a significant difference between the effect of GL, G and FL, F at the same ED level. SFE: specific fructose effect (relative difference between the isocaloric FL, F and GL, G groups).

Relative to the carbohydrate-free-high-fat L_6.03_ diet, the weight of the kidneys increased linearly with the F levels in the diets, and it was always higher in the FL and F groups than in the corresponding GL and G controls (Table 4 and Figure 8). A significant F effect was only at the highest carbohydrate level (Table 4 and Figure 8).

Kretowicz et al. [37] reported that F and sucrose may induce renal hypertrophy and tubulointerstitial disease in rats. Altunkajnak et al. [38] found increased kidney volume and histopathological renal deformities in rats on a high-fat diet.

Nakagawa’s hypothesis [39] may be the explanation for the kidney’s weight gain, i.e., in the kidney, F is predominantly metabolized in the proximal tubules; however, when present in excess, F likely becomes deleterious, possibly due in part to excessive uric acid, which is a by-product of F metabolism.

A potential mechanism is that uric acid suppresses aconitase in the Krebs cycle and therefore reduces mitochondrial oxidation. Consequently, F favors glycolysis over mitochondrial respiration, a process that is similar to the Warburg effect in cancer cells.

#### 3.4.5. Weight of Spleen

Earlier, we found that replacing F for starch in an AIN-93G type rat diet (65% F) significantly increased the weight of the spleen [11].

In the present experiment, only in rats on the FL_4.28_ and F_3.85_ diets were spleen weight slightly or significantly higher (5.3% and 15.9%, respectively) than in rats on the carbohydrate-free L_6.03_ diet.

**Figure 9:**
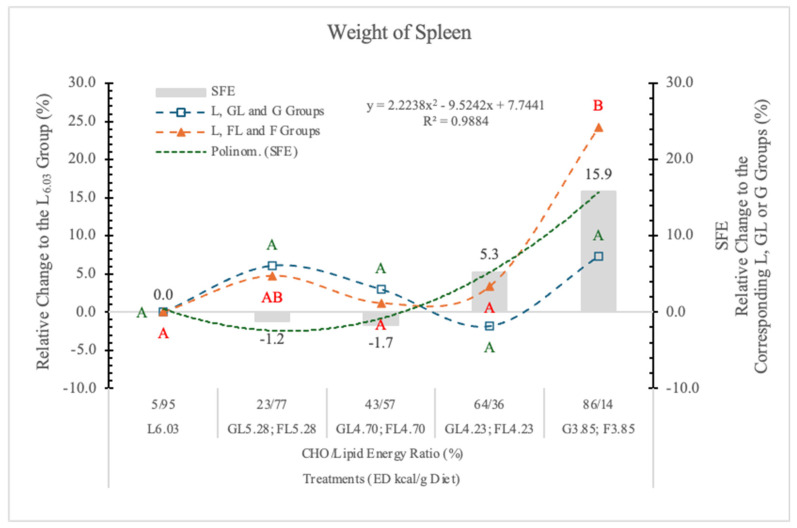
Effect of G or F on the weight of the spleen of rats fed different ED diets. Different capital letters show a significant difference among the groups consuming diets with the same carbohydrate. SFE: specific fructose effect (relative difference between the isocaloric FL, F and GL, G groups).

The difference between the G_3.85_ and F_3.85_ groups was close to the statistically significant level (*p* = 0.11) (Table 4 and Figure 9).

The high dietary levels of F may influence the gut microbiota, resulting in increased intestinal permeability, lipopolysaccharide (LPS) absorption, and the development of metabolic syndrome [40].

### 3.5. Chemical Composition of Liver

#### 3.5.1. Liver Ash

The liver ash consists of macro and micro elements, and their metabolism may be involved in the pathogenesis of alcoholic [41] and non-alcoholic fatty liver disease [42] (NAFLD), a systemic disorder of energy, G, and lipid homeostasis.

In this experiment, the ash content of the liver does not show any consequent changes according to the type of carbohydrates or the ED of the diets. Only in rats on the carbohydrate-free L_6.03_ diet was the ash level significantly lower than in animals on the monosaccharide-containing diets (Table 5).

#### 3.5.2. Liver Protein

Compared to the L_6.03_ group, neither the F content nor the ED of diets influenced the *protein* contents of the liver significantly (Table 5).

#### 3.5.3. Liver Fat (Ether Extract)

Schwarz et al. [43] demonstrated that the “de novo” lipogenesis was ~60% higher in healthy men fed an isocaloric, high-F diet (25% of the calories) compared to subjects on a complex carbohydrate diet. The high-F diet was associated with both higher lipogenesis and higher liver fat content in all participants.

**Figure 10:**
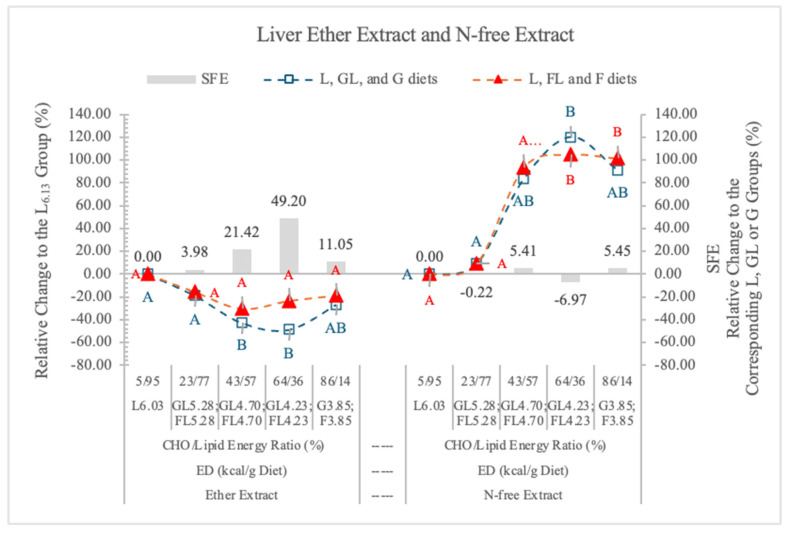
Effect of G or F on the ether extract and N-free extract of the liver of rats fed different ED diets. Different capital letters show a significant difference among the groups consuming diets with the same carbohydrate. SFE: specific fructose effect (relative difference between the isocaloric FL, F and GL, G groups).

In the present experiment, relative to the carbohydrate-free L_6.03_ diet, the decreasing ED diets reduced the lipid content of the liver, and the differences were significant at the GL_4.70_ and GL_4.23_ dietary groups. However, in the FL_4.70_, FL_4.23_, and F_3.85_ dietary groups, the liver lipid contents were higher than in the corresponding isocaloric GL and G groups (21.4%, 48.2%, and 11.05%, respectively) (Table 5 and Figure 10), and the SFE was not significant. The difference between the effects of the two monosaccharides on the liver fat content also was not significant.

We suggested that the different magnitudes of the St, G, or F effect on the liver lipid content may be explained by the differences in the rapidity of intestinal absorption of starch and the monosaccharides, and in the difference between the hepatic metabolism of G and F.

Blakemore et al. [44] presented biochemical and morphological evidence that the GLUT5 transporter is expressed in the basolateral membrane of the human intestine, suggesting that the non-phosphorylated F can easily pass through the enterocytes and reach the liver.

Fallon and Kemp [45] investigated the hepatic lipid synthesis in liver homogenate and concluded that diets high in carbohydrates increased triglyceride synthesis, and starch was less effective than G, sucrose, or F; corn oil did not alter the triglyceride synthesis of hepatocytes.

It is well documented that dietary F, sucrose, and high-F corn syrup tend to induce fatty liver; however, it was also suggested that for the development of fatty liver longer period of time (8–24 weeks) was needed [46]. However, it has been reported [47] that a non-negligible fraction of fructose is able to escape splanchnic extraction in humans, F mainly (at least 70%) metabolized in the liver, where it promotes the synthesis of fat, G can be metabolized in practically all cells, consequently G intake means lower metabolic burden on the liver than the same amount of F.

It is also important, that the metabolism of F is not controlled by hormones or allosteric mechanisms. F bypasses the rate-limiting step of glycolysis catalyzed by phosphofructokinase-1.

Figure 10 shows the SFE on the lipid content of liver. The highest SFE was at 4.23 kcal/g dietary ED, where 64% to 36% was the carbohydrate energy to lipid energy ratio. At 5.28 kcal/g dietary ED (less than 23% F in the diet), the low F content of diets likely limited the SFE, although the fat level of liver was high (Table 4).

This can be explained based on the result of Jang et al. [28], that the fate of orally administered F depends on its consumed quantity. Administered in low dose (<0.5 g/kg BW), F was ~90% cleared by the intestine; only trace amounts of F, but extensive F-derived G, lactate, and glycerate were found in the portal blood.

Therefore, we surmise that in the case of low dietary concentration, the effect of F on the liver lipogenesis may be similar to that of G (Figure 10).

Johnston et al. [48] found that there was no difference between high-F and high-G diets on liver triacylglycerol in healthy overweight men.

Sun and Empie [49] reviewing isotopic tracer studies concluded that “although F is a potent lipogenic substrate, the observed fat synthesis from F carbons is quantitatively minor compared with other pathways of F disposal. Only 0.05% and 0.15% of F were converted to de novo fatty acids and TG-glycerol at 4 h, respectively”.

What is the origin of the hepatic lipids in rats on high-F diets remains to be answered.

According to Liao et al. [50] “dietary protein, rather than carbohydrates or fat, is the primary nutritional risk factor for metabolic liver disease in humans. Ex vivo tracing studies identified amino acids as a major carbon source for the tricarboxylic acid (TCA) cycle and lipogenesis in isolated mouse hepatocytes. In vivo, dietary amino acids are twice as efficient as G in fueling hepatic fatty acid synthesis.”

Therefore, we surmise that the origin of substrates for hepatic lipid synthesis and accumulation in animals on high-G or F diets is not the same.

The liver may get fatty acids from three sources: uptake of FFAs from blood, chylomicron remnant uptake, and de novo lipogenesis.

Hepatic de novo lipogenesis (DNL) is produced mainly from carbohydrate catabolism. DNL is believed to contribute to the pathogenesis of non-alcoholic fatty liver disease, associated with the metabolic syndrome and consequent insulin resistance [51]. High G and insulin have also been shown to inhibit fatty acid oxidation [52].

In overweight men who were on a high-F or on a high-G diet, no significant changes in hepatic concentrations of triglycerides developed; however, in the hypercaloric period, both high-F and high-G diets produced significant increases in these parameters without any significant difference between the two groups.

This indicates an energy-mediated, rather than a specific macronutrient-mediated, effect [48].

The uptake of G by the hepatocytes is mediated through the GLUT2 transporter, which does not require insulin for activation.

It is expressed in the liver, intestine, kidney, and pancreatic islet beta cells and is required for G-stimulated insulin secretion [53].

G is essential for insulin-stimulated lipid synthesis; however, lipogenesis in the absence of insulin does not need G [54].

It is a good substrate for hepatic lipid synthesis, and with and without exogenous insulin, significantly decreased leucine, valine, threonine, isoleucine, phenylalanine, and tyrosine in plasma concentration [55].

Hexoses serve only indirectly, providing precursors (acetyl-CoA, lactate, NADPH, glycerol 3-phosphate) for this process.

Contrary to the effect of G, in the high F-exposing group, amino acids of arginosuccinate, ethanolamine, aspartic acid, and glutamic acid were elevated significantly, while serum creatinine and amino acids of 1-methyl-l-histidine, phosphorylethanolamine, arginine, glutamine, gamma-aminobutyric acid, amino-adipic acid, 5-hydroxylysine, and cystine decreased significantly [56].

F in the hepatocytes bypasses the gating step of glycolysis, yielding precursors for gluconeogenesis and de novo lipogenesis. It also rapidly depletes the intracellular adenylate energy charge (AEC) (ATP + 0.5ADP + ATP + ADP + AMP), resulting in increased uric acid production due to activation of AMP deaminase [57]. The F-induced depletion of liver adenine nucleotides inhibited protein synthesis [58]; consequently, the apoprotein synthesis may also be inhibited, and the ineffective packing of triglyceride into nascent VLDL may be a factor in the pathomechanism of F-induced liver steatosis.

On this basis, we surmise that in the F-fed animals, the amino acids mobilized from the peripheral tissues can also provide substrates for the hepatic lipid synthesis. The rapid metabolism of F results in uric acid release [59] from the liver, which can stimulate glucocorticoid and glucagon secretion [11]. These two hormones mobilize amino acids and fatty acids from the peripheral tissues and provide substrates for hepatic lipid synthesis and accumulation. Therefore, we hypothesize that not only F-derived precursors are the substrates for the hepatic lipid accumulation in rats on the high F diets.

Based on our previous and present experimental data, we conclude that compared to starch (complex carbohydrate), the monosaccharides, especially G and F, significantly increase the fat content of the liver. If G is the control carbohydrate, no significant F effect can be detected, despite that higher dietary monosaccharide levels were fed.

The lipid content of the liver was always higher in rats on FL and F diets than in the corresponding GL and G groups (Figure 10).

We surmise that the high lipid accumulation in the liver is the consequence of the “de novo” lipid synthesis accelerated by the precursors derived from the rapidly absorbed monosaccharides (G or F), and in the case of high F diets, the amino acids mobilized from the peripheral tissues. The magnitude of lipid accumulation may depend on the type of carbohydrate, the actual ED of diets, and the length of the experiment.

The most important factor of lipid accumulation in the liver is the very high L content (45%) of the diet.

According to Ferramosca et al. [60], high-fat diets strongly inhibited hepatic lipogenesis, resulting in an accumulation of triglyceride in the liver, so the FFA and chylomicron uptake may be the primary cause of lipid accumulation. In an earlier experiment, we showed that poor-quality protein and a deficiency of essential fatty acids in the diets of cats caused fatty liver syndrome [61].

#### 3.5.4. Liver N-Free Extract (NfE) Glucose and Glycogen)

Nitrogen-free extract (NfE) represents the carbohydrate content of the liver, such as G and glycogen. It is a calculated parameter, the difference between the weight of dry matter and the sum of the weights of ether extract, crude protein, and ash.

In the present experiment, there was no significant difference in the percentage of NfE contents of the liver of rats on GL, G or FL, F diets, at the studied ED levels (Table 5, Figure 10). When the proportion of carbohydrate to lipid energy was 23/77% in the diet, the NfE contents of the liver were as low as in the rats on the carbohydrate-free L_6.03_ diet (Figure 10). There are negative correlations between the ether extract and NfE contents of liver in both monosaccharide groups (G groups r = −0.896; F groups r = −0.797). From the G and glycogen contents of the liver, there is no difference between the effects of the two monosaccharides. F does not increase the hepatic G and glycogen storage more than G.

Rémésey et al. [62] conditioned rats to 42 or 79% carbohydrate-containing diets and found that the G concentration in the portal blood was 8.8 mM and the net hepatic output of G was still positive. Animals on the 79% carbohydrate diet exhibited hepatic G uptake, and this was at the expense of a much higher G concentration (13.6 mM) in the portal blood.

### 3.6. Metabolomic Indices

#### 3.6.1. Serum Glucose

The blood G level in the healthy rats is in the range between 5.65 and 7.90 mmol/L [63]. If we assume a 40% hematocrit level [64], this value corresponds to 9.4 ± 2.72 and 13.2 mmol/L serum G concentrations.

**Figure 11:**
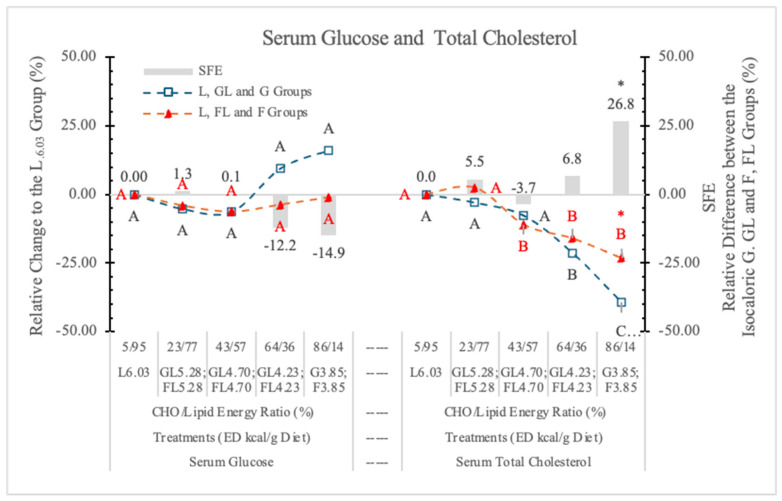
Effect of G or F on the serum glucose and total cholesterol level of rats fed different ED diets. Different capital letters show a significant difference among the groups consuming diets with the same carbohydrate. The star (*) shows a significant difference between the effect of GL, G and FL, F at the same ED level. SFE: specific fructose effect (relative difference between the isocaloric FL, F and GL, G groups).

The postprandial serum G concentrations of rats in the current study were in the normal range (7.91 ± 0.68 and 9.82 ± 3.11 mmol/L) (Table 6 and Figure 11). However, high monosaccharide dietary concentrations (86/14% and 64/36% carbohydrate to lipid energy ratios) resulted in 14.9 and 12.2% lower serum G levels in rats on the FL or F diets than in the corresponding isocaloric GL or G dietary groups (Figure 11). However, these differences were not significant.

Jang et al. [28] reported that the small intestine shields the liver from otherwise toxic F exposure. Others [65] reported that F can significantly (*p* < 0.05) decrease fasting and postprandial blood glucose compared to G.

#### 3.6.2. Serum Total Cholesterol (STCh)

Table 6 and Figure 11 show that decreasing dietary ED (replacing carbohydrate for L content of diets) significantly decreased the STCh concentrations in both monosaccharide groups; however, in the case of the L-free G_3.85_ and F_3.85_ groups, the difference between them was significant. This difference may be attributed to the cholesterol-increasing effect of F [7].

In our earlier experiment [11], replacing G with starch in the diet significantly depressed the STCh level in rats. This cholesterol-lowering effect was not observed when substituting F for starch.

These results confirmed the finding of Mamikutty et al. [66] that giving 20% F-containing drinking water did not change the STCh level in the plasma of rats. However, the data from this experiment and the results of Jameel et al. [67] suggest that the administration of F as a sole source of energy modulates plasma lipids and increases the HDL and LDL cholesterol.

We conclude that F (in high dietary concentration) and L (according to the change of the ED of diets) increases the STCh concentration.

#### 3.6.3. Serum Triglyceride (STG)

In our earlier study [11], where starch was the carbohydrate in the control diet, both the G_3.85_ and the F_3.85_ diets increased, while the carbohydrate-free L_6.03_ diet decreased the serum TG level significantly (*p* < 0.05).

**Figure 12:**
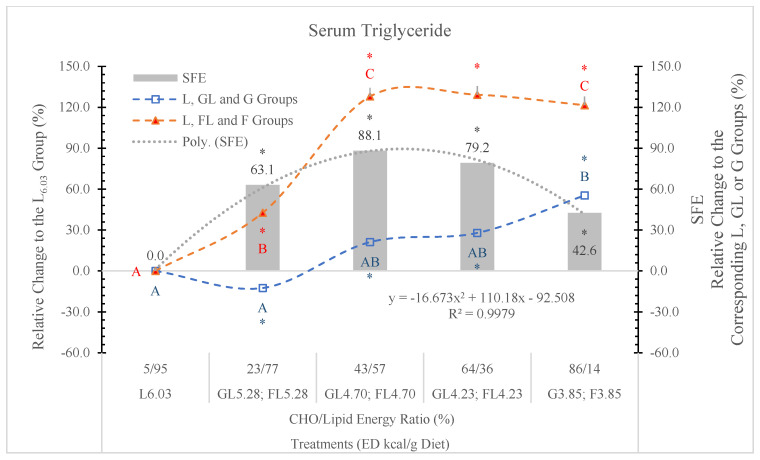
Effect of G or F on the serum triglyceride level of rats fed different ED diets. Different capital letters show a significant difference among the groups consuming diets with the same carbohydrate. The star (*) shows a significant difference between the effect of GL, G and FL, F at the same ED level. SFE: specific fructose effect (relative difference between the isocaloric FL, F and GL, G groups).

In the current experiment, low G level (23%) in the diet did not influence the STG concentration, but at higher G levels, the diets increased STG. (Table 6 and Figure 12).

STG levels in rats fed the FL and F diets were always significantly higher than in the GL or G dietary groups (Figure 12).

According to Byers and Friedman [68], the liver is the major source of plasma TG. In the hepatocytes, after the phosphorylation of F on the first carbon, it bypasses the rate limiting step of the glycolytic pathway (catalyzed by phosphofructokinase) and is metabolized directly into triose phosphate, perturbs G metabolism and G uptake pathways, and leads to a significantly enhanced rate of de novo lipogenesis and triglyceride synthesis driven by the high flux of glycerol and acyl portions of TG molecules from F catabolism [69].

#### 3.6.4. Triglyceride-Glucose Index (TyG)

In fasting conditions, the TyG is a good measure that incorporates triglyceride and G levels and serves as an effective biomarker for insulin resistance and may serve as a significant predictor for the advancement of pre-diabetes to T2DM [70]. Angoorani et al. [71] also suggest that the high TyG value predisposes to metabolic syndrome.

Because F feeding predisposes the animals to diabetes and NAFLD, we surmise that the TyG index can also be a good predictor of these metabolic disorders.

**Figure 13:**
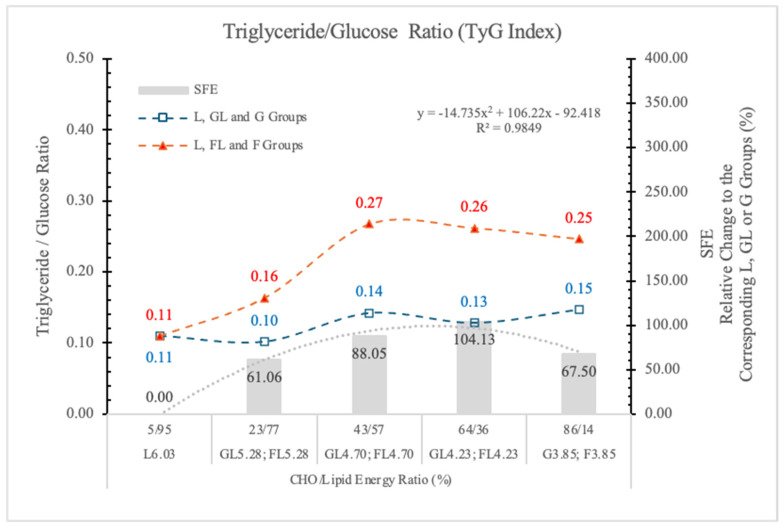
Triglyceride–glucose index (TyG) in rats fed different ED diets. SFE: specific fructose effect (relative difference between the isocaloric FL, F and GL, G groups).

Figure 13 shows that compared to the GL and G counterparts, the TyG index is significantly increased in rats on the isocaloric FL or F diets. The SFE shows dose-related changes.

We conclude that the TyG index can be a good predictor of risk assessment for diabetes or NAFLD in both humans and animals.

The F effect on the STG level depended on both its concentration in the diet and the ED of the diets, and the most pronounced effect was in animals on the FL_4.73_ and FL_4.23_ diets. The dose SFE effect was polynomial (Figure 13).

This result is in agreement with the data of Kazumi et al. [72], F stimulates triglyceride production but impairs triglyceride removal, whereas G stimulates both of them.

#### 3.6.5. Lactate Dehydrogenase (LDH)

LDH is present in almost all tissues, but at high concentrations in muscle, liver, and kidney. Its function is to catalyze the reversible conversion of lactate to pyruvate with the reduction of NAD^+^ to NADH and vice versa [73].

In the hepatic metabolism of F, the last steps are the conversion of pyruvate to acetyl-CoA or alternatively via the effect of LDH into lactate. This is why serum LDH activity is a good tracer of F-induced liver injury.

Therefore, if F-feeding is hepatotoxic, the plasma LDH activity should be increased. Indeed, Puri et al. [74] provided evidence in favor of the possibility that dietary F is associated with hepatotoxicity.

**Figure 14:**
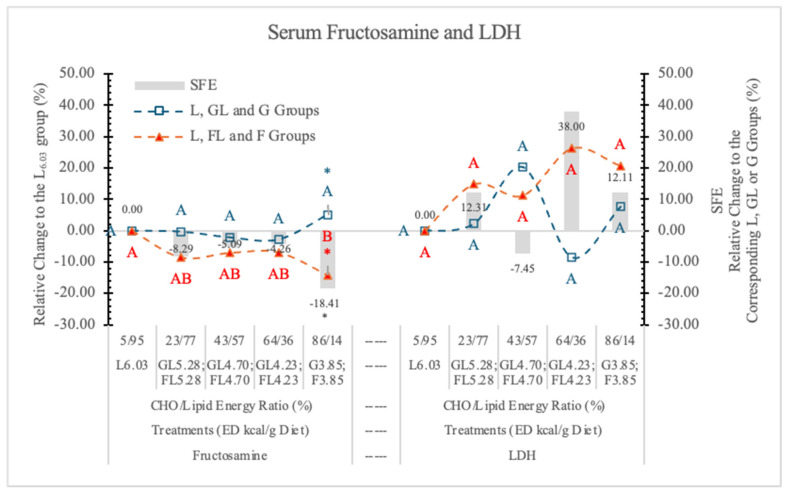
Effect of G or F on the serum fructosamine and lactate dehydrogenase (LDH) level of rats fed different ED diets. Different capital letters show a significant difference among the groups consuming diets with the same carbohydrate. The star (*) shows a significant difference between the effect of G or F at the same ED level. SFE: specific fructose effect (relative difference between the isocaloric FL, F and GL, G groups).

In this experiment, in rats on the two highest F diets (FL_4.23_ and F_3.85_) the LDH activities were higher than those of rats on the corresponding GL or G diets, but the differences were not significant (Table 7 and Figure 14).

It is possible that in a longer experiment, the high F doses would induce a significant increase in LDH activity. Serum LDH did not show correlation with any of the studied parameters. There was no significant SFE on the serum LDH concentration (Figure 14).

#### 3.6.6. Fructosamine

Fructosamine determines the glycated fraction of total serum proteins (albumin, globulins, and lipoproteins).

Because albumin is the most abundant of the serum proteins, fructosamine is predominantly a measure of glycated albumin and reflects one- to two-week changes in blood G. Fructosamine has a potential role in the diagnosis, monitoring, and management of diabetes [75,76].

Table 7 and Figure 14 show that fructosamine levels were lower in rats on FL and F vs. GL and G diets at each ED level. However, the difference between the two monosaccharide groups was significant only at the highest dietary carbohydrate concentration (3.85 kcal/g dietary ED).

According to Levi et al. [77], blood F, cholesterol, fructosamine, and glycated hemoglobin levels, and urine lipid peroxidation products were significantly higher in F-fed rats compared with the other sugar-fed and control rats.

The low fructosamine level in individuals without diabetes was found to be associated with increased mortality [78]. A proof of SFE on decreasing fructosamine levels is that there was a significant difference between the F_3.90_ and the high-fat L_6.13_ diets (Table 7 and Figure 14).

### 3.7. Effect of Glucose (G) or Fructose (F) on Hormones

#### 3.7.1. Serum Insulin

It is well documented that, contrary to G, long-term F-feeding may result in impaired insulin action in the liver and peripheral tissues [79] in both healthy and diabetic subjects. F produced a smaller postprandial rise in plasma G and serum insulin than the other carbohydrates [80].

**Figure 15:**
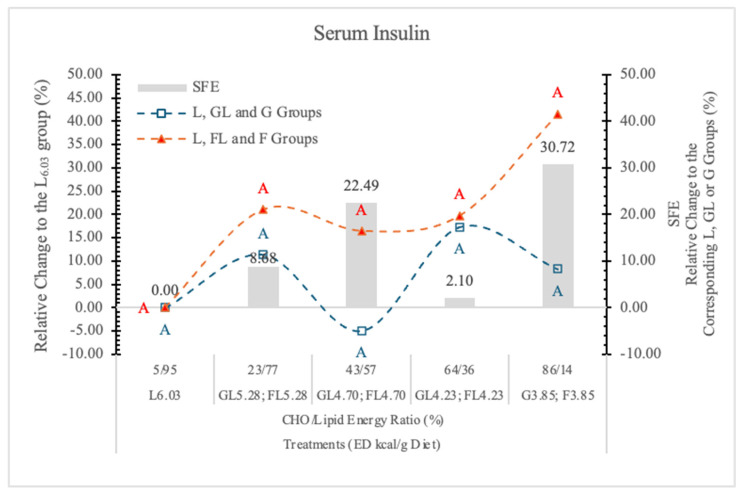
Effect of G or F on the serum insulin level of rats fed different ED diets. Different capital letters show a significant difference among the groups consuming diets with the same carbohydrate. SFE: specific fructose effect (relative difference between the isocaloric FL, F and GL, G groups).

In the present experiment, there was no significant difference between the two monosaccharide groups at any dietary ED levels; however, at the highest dietary F concentration, the serum insulin concentration was 39.6% higher than it was in rats on the G diets (Figure 15).

Long-term F consumption induces insulin resistance, impaired G tolerance, hyperinsulinemia, hypertriglyceridemia, and hypertension in animal models [66]. In case of lower dietary F concentration, the effect of F on insulin secretion may be similar to that of G, because most of the absorbed F is converted to G in the small intestinal mucosa [45].

#### 3.7.2. Serum Glucagon

Glucagon is a regulator of hepatic G production in vivo during fasting, exercise, and hypoglycemia [81]. Glucagon secretion is inhibited by G, although certain amino acids such as arginine stimulate it [82]. The interaction between the pancreatic α- and β-cells has been proven important for G homeostasis in vivo [83].

The effect of F on glucagon secretion is not clear, although Goto et al. [84] reported that F inhibited the glucagon and stimulated the insulin responses to arginine in the isolated rat pancreas.

In our earlier experiment [11], replacing starch with G decreased and with L significantly increased the serum glucagon level.

In the present experiment, in rats on the GL and G diets, the serum glucagon levels were significantly lower than in the carbohydrate-free L_6.03_ group at each dietary ED levels.

A low dose of F (23% in the diet), similar to G, also significantly decreased the serum glucagon concentration.

**Figure 16:**
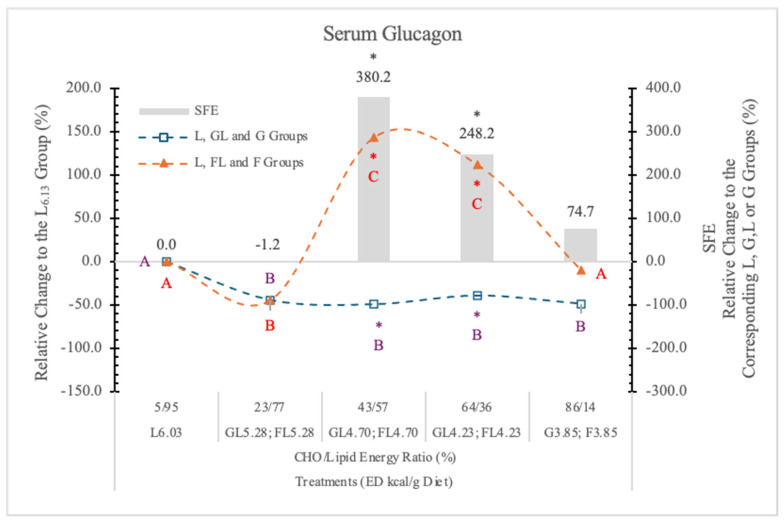
Effect of G or F on the serum glucagon level of rats fed different ED diets. Different capital letters show a significant difference among the groups consuming diets with the same carbohydrate. The star (*) shows a significant difference between the effect of GL, G and FL, F at the same ED level. SFE: specific fructose effect (relative difference between the isocaloric FL, F and GL, G groups).

In the FL_4.73_ and FL_4.23_ groups, where the F to L derived energy ratio was 43/57% and 67/33% in the diet, the serum glucagon level (*p* < 0.05) increased significantly (248.2% and 380.2%, respectively) over the glucagon concentrations in rats on the isocaloric GL_4.70_ and GL_4.23_ diets (Table 7 and Figure 16). The very high glucagon concentration in the rats on the FL_4.73_ and FL_4.23_ diets suggests a strong synergistic stimulatory effect of F with L.

The fact that the serum glucagon level in the F_3.85_ group was just slightly higher than it was in the L_6.03_ group (Figure 16) suggests that F itself is not as effective in increasing serum glucagon level as it is in combination with L. This is an important observation for understanding the physiological and pathophysiological effects of food interactions.

Based on these data, it is clear that the effect of F on the plasma glucagon level varies with the levels of F and L in the diets.

The mechanism/s of effect/s of this F and L combination on the glucagon secretion are not clear. However, at higher F concentrations, F rapidly reaches the liver, and its rapid metabolism depletes the energy level of the hepatocytes and also the pancreatic α-cells. These cells have GLUT5 transporters [85] and KHK enzyme [86]. Therefore, they can rapidly phosphorylate F, which may deplete the cytosolic ATP pool, resulting in an energy deficit, which can trigger glucagon release.

The different saturated long-chain fatty acids, especially palmitic and stearic acid, that are present in large proportions in L, also stimulate the glucagon secretion of the pancreatic α-cells [87]. Another possible explanation is that insulin resistance, which caused by the excessive consumption of F or sucrose [88,89], stimulates glucagon secretion, resulting in glucagon hypersecretion [90].

The exact effects of lipids on plasma glucagon concentration are unclear, since both inhibitory and stimulatory effects have been reported by others [91,92]. Most likely, the different environmental conditions, types and quantity of carbohydrates in the diets, and fatty acid composition of the used lipids [84] may explain some of these opposing findings.

#### 3.7.3. Serum Glucagon-to-Insulin Ratio (G/I)

Glucagon and insulin regulate carbohydrate and lipid metabolism, and they work together to control the blood sugar level. Because these two hormones have opposing effects on carbohydrate and lipid metabolism, it makes sense to consider the glucagon-to-insulin ratio (G/I ratio) as a predictor for the risk of metabolic diseases such as diabetes and NAFLD, instead of assessing each absolute value separately. Moh et al. [93] supported the idea that a lower glucagon-to-insulin ratio may be independently associated with NAFLD in participants with Type 2 Diabetes. Bang et al. [94] also reported that the fasting glucagon-to-insulin ratio is inversely associated with metabolic syndrome in patients with type 2 diabetes.

**Figure 17:**
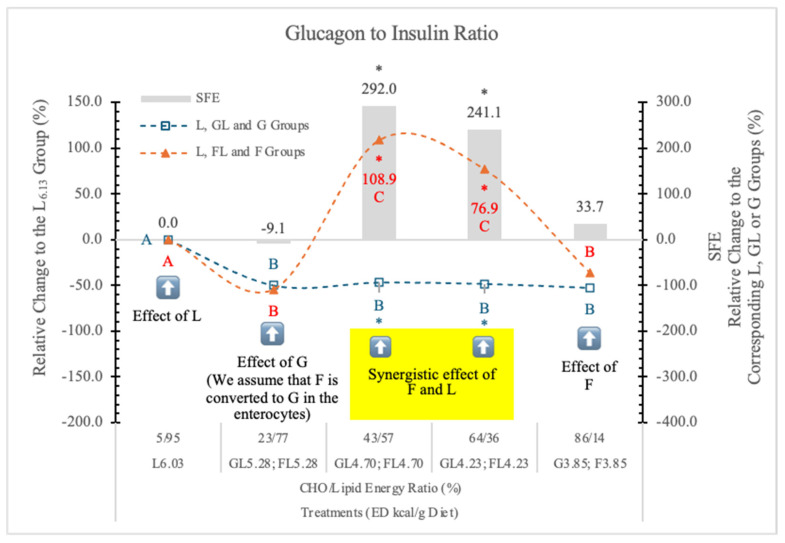
Effect of G or F on the serum glucagon to insulin ratio of rats fed different ED diets. Different capital letters show a significant difference among the groups consuming diets with the same carbohydrate. The star (*) shows a significant difference between the effects of GL, G and FL, F at the same ED level. SFE: specific fructose effect (relative difference between the isocaloric FL, F and GL, G groups). The yellow colour indicates that there is a synergy between the effects of F and L at these two ED values.

Diets high in F can rapidly produce all the key features of the metabolic syndrome [95]. Figure 17 shows the relative change of the glucagon-to-insulin ratio in rats on different experimental diets.

Compared to the carbohydrate-free L_6.03_ group, the glucagon-to-insulin ratio was lower in the GL_5.28_ and FL_5.28_ groups. Further, the decreasing ED or the increasing G levels in the GL_4.70_, GL_4.23_, and G_3.85_ diets did not induce any further changes.

However, in the FL_4.70_ and FL_4.23_ dietary groups glucagon-to-insulin ratio significantly increased, but in rats on the F_3.85_ diet, it decreased to the level in the G_3.85_ group. The glucagon-to-insulin ratio did not show any significant correlation with the lipid contents of the diets. Significant synergistic F and L effect can be seen in the case of FL_4.70_ and FL_4.23_ groups.

We conclude that the effect of F on the G/I ratio depends on the F to lipid ratio. It is not significant (−9.1%) at 23 to 77%, very high at 43 to 57% and 64 to 36% (+291 and 241%), and low and not significant again (+34%) at 86 to 14% F to lipid energy ratios.

#### 3.7.4. Serum Leptin

Jian-Mei et al. [96] reported that F consumption significantly increased the serum leptin and uric acid levels in rats by upregulating the expression of obesity genes in adipose tissue. Others [97] reported that chronic F consumption caused leptin resistance, while serum leptin levels, weight, and adiposity were the same as in the leptin-responsive control rats. The replacement of starch with G or L in the feed decreased the postprandial serum leptin concentration [11].

Teff et al. [98] suggest that chronic consumption of a high F diet decreases the circulating insulin and leptin levels, and increases ghrelin concentrations, which could lead to increased caloric intake and contribute to weight gain and obesity.

Shapiro et al. [97] reported that “chronic fructose consumption induces leptin resistance prior to body weight, adiposity, serum leptin, insulin, or G increases, and this fructose-induced leptin resistance accelerates high-fat induced obesity”.

**Figure 18:**
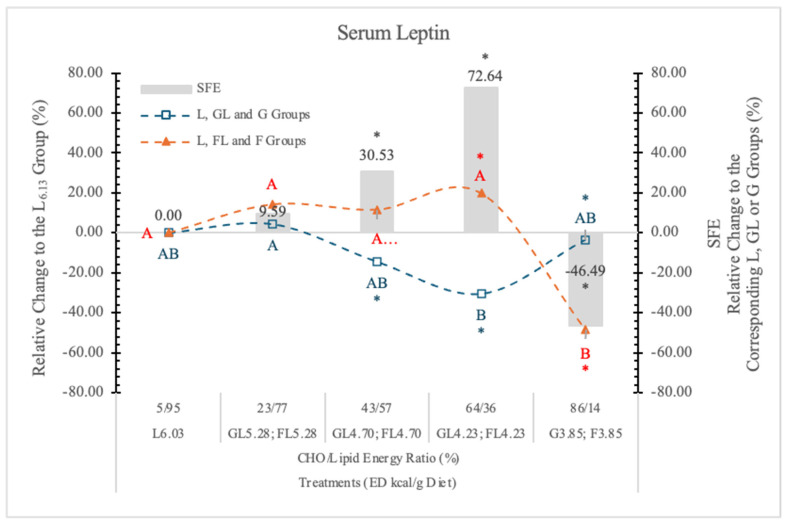
Effect of G or F on the serum leptin level of rats fed different ED diets. Different capital letters show a significant difference among the groups consuming diets with the same carbohydrate. The star (*) shows a significant difference between the effect of GL, G and FL, F at the same ED level. SFE: specific fructose effect (relative difference between the isocaloric FL, F and GL, G groups).

In the present study, the L_6.03_ dietary group with increasing dietary concentration of G (GL_4.73_ and GL_4.23_ groups) gradually decreases the serum leptin concentration (Table 7, Figure 18). Contrary to this, F does not induce significant changes in the corresponding FL groups. In rats on the lowest ED, L-free dietary groups, the effects of F and G are exactly the opposite of what we have seen in rats on higher ED diets.

We found that in rats on a high F diet, the postprandial insulin and glucagon concentrations were higher and the leptin level lower than in the animals on the isocaloric high G diet. Increasing the dietary ED by replacing L for F, we can see the opposite effect. The leptin and glucagon concentrations were significantly higher than in the animals on the isocaloric G diets.

Based on these data, we may conclude that both the ED and the monosaccharides (G or F) in the diets significantly influence the serum leptin concentration when the carbohydrate level in the diets is more than 25% (Table 7 and Figure 16).

The feeding of F_3.85_ (lard free) diet resulted in significantly lower serum leptin concentration than it is in the G_3.85_ group. At lower F and higher dietary ED levels (FL_4.23_ and FL_4.73_ groups), this inhibitory effect disappeared, and significantly higher leptin concentrations were noted than in the corresponding isocaloric GL groups.

The direction of the SFE may be positive (at 64/36% and 43/57% F to L energy ratio; in the FL_4.73_ and FL_4.23_ groups) or negative (−46.5%, *p* < 0.05 in F_3.85_ group), depending on the energy density (L content) and the F concentration of the diets.

Table 8 summarizes the results of our previous (starch was the carbohydrate in the control diet) and present experiments (glucose is the carbohydrate in the control diets), indicating the significant differences with bold numbers.

When a starch-containing diet was the control, generally, the direction of the effects of G and F were very similar; mainly, quantitative differences could be seen. Only in the case of three parameters, the serum fructosamine, glucagon, and leptin did we see opposite effects of G and F. When the feed intake does not exceed the maintenance energy requirement, there is not too much difference between the effects of G or F. Above the maintenance dietary energy level (present experiment), both G and F also increase the fat content of the liver, but the difference between the effects of these two monosaccharides was not significant. This suggests that G almost as efficiently stimulates the lipid synthesis of hepatocytes as fructose. A significant SFE effect was detected in the case of eight parameters. These were the BWG, the weights of viscera and EWAT, the weight of liver and kidney, the fat content of liver, the serum glucagon and leptin concentrations. This also shows that intake of F or G above the maintenance energy requirements is detrimental. When comparing F and G, F has more negative effects.

**Figure 19:**
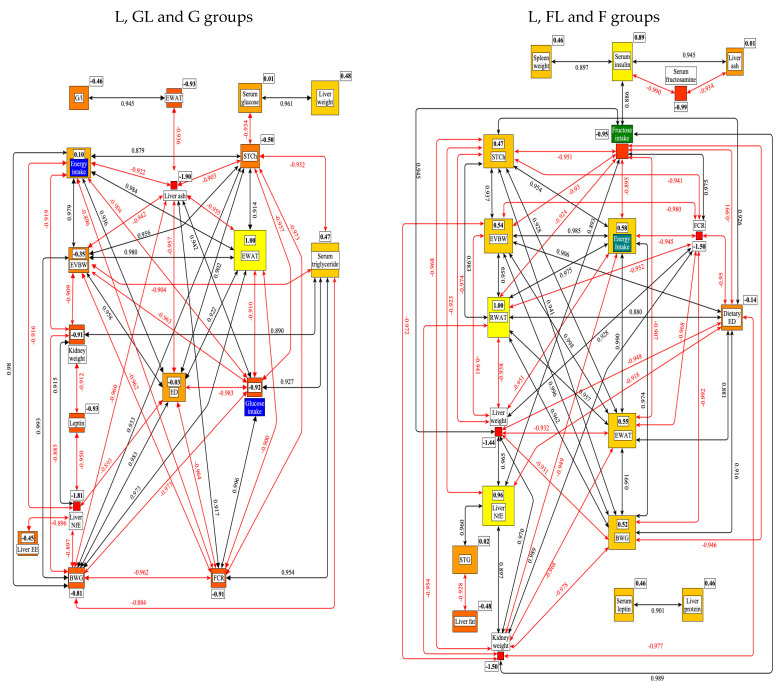
Results of the correlation and centrality (weight of the connected edges) analysis of data from rats on the L, GL, G or L, FL, F diets. BWG: body weight gain; EVBW: eviscerated bodyweight; G/I: glucagon to insulin ratio; EE: ether extract; NfE: nitrogen free extract; EWAT: eviscerated white adipose tissue; RWAT: retroperitoneal white adipose tissue; FCR: feed conversion rate; ED: energy density; STCh: serum total cholesterol; STG: serum triglyceride. The framed numbers and the colors (yellow: the most central; red: the least central) show the positive or negative weight of a given parameter in the network.

Figure 19 shows the significant correlations among the tested parameters and the results of the centrality analysis (weights of the connected edges) in the metabolic network.

This figure shows that in the case of G groups, the RWAT, and in F groups, the EWAT proved to be the most central nodes in the network. These two white adipose tissues may be of central importance in understanding the SFE. This result indicates that adipose tissues play an important role in regulating energy and G homeostasis, acting as an endocrine organ, producing bioactive factors such as adipokines [99].

Further evidence for the significance of EWAT and/or RWAT in the pathology of obesity is that the denervation of RWAT improved the cardiometabolic dysfunctions in a high-fat animal model [100]. Another important conclusion can be that in most of the studied parameters; only quantitative differences could be detected between the effects of G and F; the direction of their effect was identical.

## 4. Conclusions

From the experimental data, the following conclusions can be drawn:

In several cases, the effects of G and F intake on the studied parameters are similar, showing negative (dietary energy density, energy intake, body weight gain, eviscerated body weight, retroperitoneal white adipose tissue, serum total cholesterol) or positive (feed conversion ratio, serum triglyceride, and liver N-free extract) correlations. In these cases, the direction of their effect is similar, only the magnitude of the effect is different.

Five parameters showed significant correlation with the F intake (liver weight, kidney weight, serum LDH, insulin, and fructoseamine) and one with the G intake (liver ash).

The most important factor influencing the feed intake of rats is the satiation effect (consumed quantity) of the diet; however, a special glucose effect is also detectable. This indicates that when the dietary energy intake exceeds the requirement (high energy density diets), glucose depresses the appetite by 6 to 10%. If the daily energy intake does not exceed the requirement, there is no significant difference between the effects of G and F on feed intake.

The energy intake is mainly determined by the energy density of the diets, and to a lesser extent by the amount of feed consumed. The appetite depressing effect of dietary glucose was also observed.

The effect of fructose on body weight gain depends on both the energy density and the actual dietary fructose level.

The fructose effect on body weight gain is the result of the organ weight change of viscera, and the direction of the effect (negative or positive) depends on both the fructose and energy density levels of the diets.

The energy density of the diets not only influences the energy consumption but also improves the feed conversion ratio, and its improvement is not glucose or fructose dependent. This result is particularly important for the understanding of the mechanism of dietary effects on obesity.

There is a polynomial relation between the fructose and/or energy density of diets and the weight of the liver. The magnitude of the specific fructose effect depends on both the energy density and the actual fructose level of diets.

Glucose and fructose influence the weight of EWAT differently. The data show that G vs. F prevent lipid accumulation in this fat tissue.

F increases the weight of the kidney and spleen more than G. This may result from increased glycogen levels in the kidney. We have no explanation for the weight gain of the spleen.

It is important to note that, contrary to starch, both glucose and fructose significantly increase the fat content of the liver, and there is no significant difference between the effects of the two monosaccharides. We suggest that the rapid absorption and the metabolic difference between glucose and fructose are the important factors in stimulating the lipid storage and synthesis in the hepatocytes.

While fructose does not influence the blood sugar level, above a certain dietary concentration (40%), glucose does.

Relative to a carbohydrate-free lard diet, both monosaccharides dose dependently decrease the serum total cholesterol level; however, in high concentrations, glucose seems to be more effective.

A specific fructose effect can be noted for increasing serum triglyceride levels. While glucose increases serum triglycerides significantly only at the highest dietary concentration, fructose does it at each dietary level.

In rats on fructose-containing diets, the serum insulin concentration was higher at any energy density levels than it was in the corresponding glucose groups, suggesting a mild risk for type 2 diabetes.

Relative to glucose, both fructose and lard alone and in combination with each other increase serum glucagon levels, and their combination is synergistic when the dietary fructose level is more than 23%.

Enterocytes convert fructose to glucose when it is present in low concentrations in the diet, and this glucose cancels the stimulating effect of fat on glucagon secretion.

The glucagon to insulin ratio clearly shows that a catabolic metabolic situation exists in rats when the fructose to lard energy ratio in the diet is somewhere 43 to 57 and 64 to 36%, despite the fact that the animals consume more energy and other nutrients than they need. This dietary concentration and ratios of F and L appear to be most detrimental to the progression of the metabolic syndrome.

The effect of F and ED on the serum leptin level can be negative or positive, depending on the dietary F concentration and the energy density of diets.

A specific fructose effect compared to glucose can be noted within body weight gain, weight of the visceral organs (liver and kidney), epididymal white adipose tissue, lipid content of liver, serum triglyceride concentration, serum glucagon, and leptin levels.

We suggest that for the negative effects (weight gain, increased risk of type 2 diabetes, heart disease, fatty liver disease, and other metabolic disorders) of sugar and fructose corn syrup, not only the fructose, but their glucose contents may also be responsible, especially when the daily energy consumption exceeds the maintenance energy requirement. Consequently, the slowly absorbable complex carbohydrates should be preferred to rapidly absorbable monosaccharides.

## Figures and Tables

**Table 1:** Percent formulation of experimental diets.

	Treatments				
	L_6.03_	GL_5.28_ or FL_5.28_	GL_4.70_ or FL_4.70_	GL_4.23_ or FL_4.23_	G_3.85_ or F_3.85_
Casein (%)	15.65	13.71	12.20	10.99	10.00
Milk protein isolate (%)	15.65	13.71	12.20	10.99	10.00
Cellulose (%)	7.83	6.86	6.10	5.50	5.00
Corn oil (%)	7.83	6.86	6.10	5.50	5.00
L (%)	45.22	29.71	17.63	7.94	0.00
G or F (%)	0.00	22.29	39.66	53.59	65.00
AIN93VX mineral premix (%)	5.48	4.80	4.27	3.85	3.50
AIN93VX vitamin premix (%)	1.57	1.37	1.22	1.10	1.00
Cyst, Met, choline (%)	0.78	0.69	0.61	0.55	0.50
Sum (%)	100.00	100.00	100.00	100.00	100.00
Calculated GE contents (kcal/100 g diet)	602.61	528.00	469.83	423.21	385.00
Calculated protein content (g/100 g diet)	31.30	27.43	24.41	21.98	20.00
Calculated protein energy ratio (g/kcal)	19.25	19.25	19.25	19.25	19.25

Letters L, G, and F stand for lard, glucose, and fructose. Subscripts show the gross energy (GE) content of diets (kcal/g).

**Table 2:** Production-related parameters (n = 8/treatment groups).

Feed Intake (g/day/100 g BW)				
L_6.03_	GL_5.28_	GL_4.70_	GL_4.23_	G_3.85_
6.35 ± 0.37 A	6.85 ± 0.36 * B	7.28 ± 1.12 BC	6.57 ± 1.03 BA	7.53 ± 0.23 C
	FL_5.28_	FL_4.70_	FL_4.23_	F_3.85_
	7.59 ± 0.72 * B	7.74 ± 0.85 B	7.20 ± 0.93 B	7.68 ± 0.89 B
Calculated G or F Intake (g/100 g BW/Day)				
L_6.03_	GL_5.28_	GL_4.70_	GL_4.23_	G_3.85_
0.00	1.11 ± 0.06	3.20 ± 0.50	2.37 ± 0.36	4.89 ± 0.15
	FL_5.28_	FL_4.70_	FL_4.23_	F_3.85_
	1.23 ± 0.12	3.51 ± 0.45	2.52 ± 0.28	4.99 ± 0.58
Calculated GE intake (kcal/day/100 g BW)				
L_6.03_	GL_5.28_	GL_4.70_	GL_4.23_	G_3.85_
38.88 ± 2.28 A	36.87 ± 1.96 * A	34.85 ± 5.36 A	28.38 ± 4.44 B	29.38 ± 0.91 B
	FL_5.28_	FL_4.70_	FL_4.23_	F_3.85_
	40.86 ± 3.88 * A	37.08 ± 4.06 A	31.08 ± 4.00 B	29.95 ± 3.46 B
BW (g)				
L_6.03_	GL_5.28_	GL_4.70_	GL_4.23_	G_3.85_
392.63 ± 16.77 A	379.16 ± 17.71 BA	365.69 ± 26.30 B	342.06 ± 22.02 BC	335.83 ± 13.63 C
	FL_5.28_	FL_4.70_	FL_4.23_	F_3.85_
	390.50 ± 25.21 A	379.44 ± 20.26 A	343.13 ± 29.98 B	326.13 ± 28.72 B
BWG (g/day/100 g BW)				
L_6.03_	GL_5.28_	GL_4.70_	GL_4.23_	G_3.85_
2.11 ± 0.49 A	1.91 ± 0.29 A	1.73 ± 0.53 AB	1.40 ± 0.37 BC	1.30 ± 0.17 C
	FL_5.28_	FL_4.70_	FL_4.23_	F_3.85_
	2.06 ± 0.34 A	1.91 ± 0.33 A	1.40 ± 0.27 B	1.17 ± 0.36 B
SFE	0.00	7.95	10.30	0.11
FCR (g/g)				
L_6.03_	GL_5.28_	GL_4.70_	GL_4.23_	G_3.85_
3.01 ± 0.60 A	3.59 ± 0.49 AC	4.20 ± 0.87 AB	4.69 ± 0.85 B	5.78 ± 0.76 D
	FL_5.28_	FL_4.70_	FL_4.23_	F_3.85_
	3.68 ± 0.35 AB	4.05 ± 0.35 B	5.13 ± 0.73 C	5.65 ± 0.79 C

G, F, and L stand for glucose, fructose, and lard. The subscripts show the gross energy (GE) densities of diets (kcal/g). BW: body weight; BWG: bodyweight gain. The different letters show significant (*p* < 0.05) differences among the different energy density (ED) groups. The star (*) indicates a significant difference between the appropriate isocaloric GL, G and FL, F groups.

**Table 3:** Effect of glucose and fructose on EVBW, viscera, EWAT, and RWAT (n = 8/treatment groups).

EVBW (g)				
L_6.03_	GL_5.28_	GL_4.70_	GL_4.23_	G_3.85_
319.99 ± 15.28 A	311.62 ± 16.67 AB	300.21 ± 20.53 B	272.94 ± 13.43 C	264.46 ± 19.17 C
	FL_5.28_	FL_4.70_	FL_4.23_	F_3.85_
	319.21 ± 22.79 A	307.66 ± 18.61 A	269.09 ± 29.76 B	259.70 ± 20.18 B
Viscera (g)				
L_6.03_	GL_5.28_	GL_4.70_	GL_4.23_	G_3.85_
72.6 ± 26.7 A	67.5 ± 22.3 A	65.5 ± 43.3 A	69.1 ± 29.5 A	71.4 ± 28.3 A
	FL_5.28_	FL_4.70_	FL_4.23_	F_3.85_
	71.3 ± 39.4 A	71.8 ± 30.1 A	74.0 ± 34.8 A	66.4 ± 43.3 A
EWAT (g/100 g EVBW)				
L_6.03_	GL_5.28_	GL_4.70_	GL_4.23_	G_3.85_
1.32 ± 0.41 A	0.92 ± 0.37 * B	1.02 ± 0.34 AB	0.88 ± 0.30 B	0.84 ± 0.28 B
	FL_5.28_	FL_4.70_	FL_4.23_	F_3.85_
	1.34 ± 0.35 * A	1.26 ± 0.38 AC	0.96 ± 0.30 BC	0.91 ± 0.26 C
RWAT (g/100 g EBW)				
L_6.03_	GL_5.28_	GL_4.70_	GL_4.23_	G_3.85_
1.48 ± 0.56 A	1.28 ± 0.45 A	1.28 ± 0.20 A	0.77 ± 0.36 B	0.76 ± 0.40 B
	FL_5.28_	FL_4.70_	FL_4.23_	F_3.85_
	1.66 ± 0.54 A	1.25 ± 0.43 AB	0.94 ± 0.31 B	0.59 ± 0.27 C

EVBW: eviscerated body weight; EWAT: epididymal white adipose tissue; RWAT: retroperitoneal white adipose tissue; G, F, and L stand for glucose, fructose, and lard. The subscripts show the energy densities of diets (kcal/g). The different capital letters show significant (at least *p* < 0.05) differences among the treatment groups. The star (*) indicates a significant (*p* < 0.05) difference between the appropriate isocaloric GL, G and FL, F groups.

**Table 4:** Effect of diets on the weight of different organs of rats (n = 8/treatment groups).

Weight of Liver (g/100 g BW)				
L_6.03_	GL_5.28_	GL_4.70_	GL_4.23_	G_3.85_
3.50 ± 0.31 A	3.19 ± 0.15 * C	3.24 ± 0.24 * AC	3.51 ± 0.38 * A	3.95 ± 0.48 B
	FL_5.28_	FL_4.70_	FL_4.23_	F_3.85_
	3.52 ± 0.33 * A	3.97 ± 0.65 * AB	4.27 ± 0.74 * B	4.08 ± 0.16 B
Weight of Kidney (g/100 g BW)				
L_6.03_	GL_5.28_	GL_4.70_	GL_4.23_	G_3.85_
0.63 ± 0.04 A	0.61 ± 0.05 A	0.65 ± 0.06 AB	0.69 ± 0.04 B	0.68 ± 0.05 * B
	FL_5.28_	FL_4.70_	FL_4.23_	F_3.85_
	0.65 ± 0.06 A	0.69 ± 0.07 AB	0.74 ± 0.06 BC	0.77 ± 0.06 * C
Weight of Spleen (g/100 g BW)				
L_6.03_	GL_5.28_	GL_4.70_	GL_4.23_	G_3.85_
0.28 ± 0.06 A	0.29 ± 0.03 A	0.29 ± 0.05 A	0.27 ± 0.03 A	0.30 ± 0.04 A
	FL_5.28_	FL_4.70_	FL_4.23_	F_3.85_
	0.29 ± 0.04 A	0.28 ± 0.03 A	0.29 ± 0.03 A	0.33 ± 0.02 B

G, F, and L stand for glucose, fructose, and lard. The subscripts show the energy densities of diets (kcal/g). The different capital letters show significant (at least *p* < 0.05) differences among the treatment groups. The star (*) indicates a significant (*p* < 0.05) difference between the appropriate GL, G and FL, F groups.

**Table 5:** Chemical composition of liver (n = 8/treatment groups).

Liver ash (% of DM)				
L_6.03_	GL_5.28_	GL_4.70_	GL_4.23_	G_3.85_
5.90 ± 1.28 A	6.55 ± 0.61 AB	6.47 ± 0.56 AB	7.00 ± 0.66 B	7.24 ± 2.24 AB
	FL_5.28_	FL_4.70_	FL_4.23_	F_3.85_
	6.74 ± 0.82 A	6.16 ± 1.17 A	6.45 ± 0.98 A	7.13 ± 1.25 A
Liver crude protein (% of DM)				
L_6.03_	GL_5.28_	GL_4.70_	GL_4.23_	G_3.85_
69.67 ± 4.12 A	72.34 ± 3.21 A	73.75 ± 3.64 A	72.75 ± 4.12 A	69.50 ± 7.78 A
	FL_5.28_	FL_4.70_	FL_4.23_	F_3.85_
	71.51 ± 3.55 A	71.16 ± 4.11 A	68.98 ± 6.96 A	67.53 ± 6.08 A
Liver ether extract (% of DM)				
L_6.03_	GL_5.28_	GL_4.70_	GL_4.23_	G_3.85_
19.86 ± 4.92 A	16.12 ± 2.94 A	11.39 ± 3.19 B	10.19 ± 1.52 B	14.53 ± 8.77 AB
	FL_5.28_	FL_4.70_	FL_4.23_	F_3.85_
	16.76 ± 4.74 A	13.83 ± 4.37 A	15.21 ± 8.09 A	16.14 ± 8.74 A
Liver NfE (% of DM)				
L_6.03_	GL_5.28_	GL_4.70_	GL_4.23_	G_3.85_
4.57 ± 3.31 A	4.99 ± 2.87 A	8.39 ± 5.24 AB	10.07 ± 5.33 B	8.73 ± 7.49 AB
	FL_5.28_	FL_4.70_	FL_4.23_	F_3.85_
	4.98 ± 3.24 A	8.84 ± 4.69 AB	9.36 ± 5.15 B	9.21 ± 5.04 B

NfE: nitrogen-free extract; G, F, and L stand for glucose, fructose, and lard. The subscripts show the energy densities of diets (kcal/g). The different capital letters show significant (at least *p* < 0.05) differences among the treatment groups.

**Table 6:** Effect of diets on serum glucose, cholesterol, and triglyceride levels (n = 8/treatment groups).

Serum glucose (mmol/L)				
L_6.03_	GL_5.28_	GL_4.70_	GL_4.23_	G_3.85_
8.45 ± 1.27 A	8.00 ± 1.04 A	7.91 ± 0.68 A	9.26 ± 3.30 A	9.82 ± 3.11 A
	FL_5.28_	FL_4.70_	FL_4.23_	F_3.85_
	8.10 ± 1.16 A	7.92 ± 0.56 A	8.13 ± 1.80 A	8.36 ± 1.51 A
Serum total cholesterol (mmol/L)				
L_6.03_	GL_5.28_	GL_4.70_	GL_4.23_	G_3.85_
2.10 ± 0.32 A	2.04 ± 0.27 A	1.94 ± 0.28 A	1.65 ± 0.17 B	1.27 ± 0.27 * C
	FL_5.38_	FL_4.70_	FL_4.23_	F_3.90_
	2.15 ± 0.23 A	1.87 ± 0.20 B	1.76 ± 0.33 B	1.61 ± 0.28 * B
Serum triglyceride (mmol/L)				
L_6.03_	GL_5.28_	GL_4.70_	GL_4.23_	G_3.85_
0.93 ± 0.21 A	0.81 ± 0.33 A *	1.13 ± 0.52 AB *	1.19 ± 0.50 AB *	1.44 ± 0.54 * B
	FL_5.38_	FL_4.79_	FL_4.32_	F_3.90_
	1.33 ± 0.49 * B	2.12 ± 0.96 * C	2.13 ± 0.84 * C	2.06 ± 0.38 * C

G, F, and L stand for glucose, fructose, and lard. The subscripts show the energy densities of diets (kcal/g). The different capital letters show significant (at least *p* < 0.05) differences among the treatment groups. The star (*) indicates a significant (*p* < 0.05) difference between the isocaloric GL, G and FL, F containing treatment groups.

**Table 7:** Effect of diets on serum LDH, fructosamine, insulin, glucagon, and leptin (n = 8/treatment groups).

Serum LDH (U/mL)				
L_6.03_	GL_5.28_	GL_4.70_	GL_4.23_	G_3.85_
1.53 ± 37 A	1.56 ± 0.45 A	1.83 ± 0.38 A	1.40 ± 0.50 A	1.64 ± 0.84 A
	FL_5.38_	FL_4.79_	FL_4.32_	F_3.90_
	1.75 ± 0.45 A	1.70 ± 0.18 A	1.93 ± 0.91 A	1.84 ± 0.78 A
Serum fructosamine (μmol/L)				
L_6.13_	FL_5.38_	FL_4.79_	FL_4.32_	F_3.90_
464.38 ± 37.64 A	463.13 ± 38.07 A	454.63 ± 33.61 A	451.50 ± 71.27 A	488.29 ± 88.00 A *
	FL_5.38_	FL_4.79_	FL_4.32_	F_3.90_
	424.75 ± 53.07 AB	431.50 ± 41.24 AB	400.00 ± 33.08 AB	398.38 ± 38.18 B *
Serum insulin (μg/L)				
L_6.03_	GL_5.28_	GL_4.70_	GL_4.23_	G_3.85_
1.94 ± 0.83 A	2.16 ± 1.29 A	1.84 ± 0.59 A	2.27 ± 1.69 A	1.97 ± 0.74 A
	FL_5.38_	FL_4.79_	FL_4.32_	F_3.90_
	2.35 ± 0.83 A	2.26 ± 0.46 A	2.32 ± 1.70 A	2.74 ± 1.85 A
Serum glucagon (μg/L)				
L_6.03_	GL_5.28_	GL_4.70_	GL_4.23_	G_3.85_
5.73 ± 2.03 A	3.19 ± 1.43 B	2.90 ± 1.59 * B	3.48 ± 2.05 * B	2.94 ± 1.90 B
	FL_5.28_	FL_4.70_	FL_4.23_	F_3.85_
	3.15 ± 0.48 B	13.94 ± 6.52 * C	13.77 ± 5.53 * C	5.14 ± 4.20 A
Glucagon to Insulin Ratio				
L_6.03_	GL_5.28_	GL_4.70_	GL_4.23_	G_3.85_
2.95 ± 0.53 A	1.48 ± 0.47 B	1.57 ± 0.40 B	1.53 ± 0.44 B	1.40 ± 0.29 B
	FL_5.38_	FL_4.79_	FL_4.32_	F_3.90_
	1.34 ± 0.20 B	6.17 ± 1.91 * C	5.22 ± 2.42 * C	1.87 ± 0.56 B
Serum leptin (ng/mL)				
L_6.03_	GL_5.28_	GL_4.70_	GL_4.23_	G_3.85_
2.71 ± 1.33 AC	2.83 ± 1.03 A	2.32 ± 0.50 AC	1.88 ± 0.47 CB	2.62 ± 0.97 AB
	FL_5.28_	FL_4.70_	FL_4.23_	F_3.85_
	3.10 ± 0.84 A	3.03 ± 0.71 * A	3.25 ± 1.45 * A	1.40 ± 0.36 * A

G, F, and L stand for glucose, fructose, and lard. The subscripts show the energy densities of diets (kcal/g). The different capital letters show significant (at least *p* < 0.05) differences among the treatment groups. The star (*) indicates a significant (*p* < 0.05) difference between the appropriate GL and FL groups.

**Table 8:** Effect of fructose on nutritional and metabolomic parameters (Percent change to the starch or glucose-containing diets; n = 8/treatment groups).

	Dietary carbohydrate energy to lipid energy ratio (%)								Significant effects (F versus G)	
	86/14	86/14	5/95	5/95	23/77	43/57	64/36	86/14		
	Treatment groups								Pooled data	SFE
	Relative change (%) Data from Szabó et al. [11]			Relative change (%) (Data from the present experiment)						
	G_3.85_ versus St_3.85_	F_3.90_ versus St_3.85_	L_6.03_ versus St_3.85_	L_6.03_ versus G_3.85_	FL_5.28_ versus GL_5.28_	FL_4.70_ versus GL_4.70_	FL_4.23_ versus GL_4.23_	F_3.85_ versus G_3.85_		
Feed intake	**−3.46**	−1.54	**−18.59**	**−15.73**	10.81	6.38	9.52	1.95	*	
Energy intake	−1.35	1.01	**30.98**	**32.34**	10.81	6.38	9.52	1.95	*	
BWG	−2.26	−12.03	**58.65**	**61.72**	7.95	10.30	0.11	−10.20		*
FCR (Feed/gain)	−1.37	11.95	**−48.63**	**−44.31**	2.13	−6.88	7.70	2.44		
EVSCBW	**−4.89**	**−6.62**	**15.07**	**20.98**	2.44	2.48	−1.41	−1.80		
Weight of viscera	5.40	−1.91	5.33	1.77	5.54	9.63	7.10	−6.94		*
EWAT	2.44	10.98	**60.98**	**57.83**	**45.80**	22.91	13.04	8.44	*	*
RWAT	−7.32	−28.05	**80.49**	**94.70**	30.11	−2.52	21.60	−21.93		
Liver weight	13.83	**23.92**	0.86	**−11.37**	**10.38**	**22.68**	**21.51**	8.76	*	*
Kidney weight	0.00	**13.24**	**−7.35**	**−7.79**	7.55	6.36	7.34	**13.28**	*	*
Spleen weight	11.11	**25.93**	3.70	−6.78	−1.21	−1.75	5.27	15.85		
Liver ash	8.55	6.90	**−11.54**	**−18.47**	3.02	−4.71	−7.75	−1.53		
Liver ether extract	**107.28**	**138.66**	**183.31**	**36.69**	3.98	21.42	49.20	11.05		*
Liver protein	**−8.00**	**−10.06**	**−7.25**	0.24	−1.14	−3.51	−5.18	−2.84		
Liver N-free extract	−24.94	−17.21	**−59.59**	**−47.68**	−0.22	5.41	−6.97	5.45		
Serum glucose	**29.55**	**10.29**	**11.48**	**−13.92**	1.25	0.05	−12.19	−14.87		
Serum total cholesterol	**−26.13**	1.26	**32.08**	**65.17**	5.52	−3.66	6.82	**26.83**		
Serum TG	**29.73**	**72.07**	**−16.22**	**−35.64**	**63.08**	**88.15**	**79.25**	**42.60**	*	*
Serum fructosamine	**7.22**	**−12.52**	**−16.22**	−4.90	−8.29	−5.09	−4.26	**−18.41**	*	
Serum LDH	−6.29	5.14	**−12.57**	−7.12	12.31	−7.45	38.00	12.11		
Serum insulin	5.91	47.31	−9.14	1.37	8.68	22.49	2.10	39.61		
Serum glucagon	**−41.91**	**26.02**	**40.44**	**94.71**	−1.20	**380.16**	**248.24**	74.75	*	*
Glucagon/Insulin ratio	−45.16	−14.79	54.57	**110.84**	−9.09	**292.01**	**241.08**	33.68	*	*
Serum leptin	**25.17**	**−40.15**	**44.93**	15.90	9.59	**30.53**	53.45	−40.15		*

Bold numbers show significant difference to St_3.85_ or G_3.85_ group; the star (*) indicates significant difference between the GL, G and FL, F groups; St: starch; G: glucose; F: fructose; L: lard.

## Data Availability

The original contributions presented in the study are included in the article, further inquiries can be directed to the corresponding author.

## List of Abbreviations

BW	body weight
BWG	body weight gain
EVBW	eviscerated body weight
ED	energy density of the diets
EE	ether extract
EWAT	epididymal white adipose tissue
F	fructose
FCR	Feed Conversion Ratio (Feed/Gain)
Fi	feed intake
G	glucose
GE	gross energy
L	lard
LDH	lactate dehydrogenase
NAFLD	non-alcoholic fatty liver disease
NfE	N-free extract
RWAT	retroperitoneal white adipose tissue
SFE	specific fructose effect
SPF	Specific-pathogen-free
St	starch
STCh	Serum total cholesterol
STG	Serum triglyceride
TyG	Triglyceride-glucose index
